# Supplementary material for: Duplication and subfunctionalisation of the general transcription factor IIIA (gtf3a) gene in teleost genomes, with ovarian specific transcription of gtf3ab
Source: PLoS One. 2020 Jan 30;15(1):e0227690. doi: 10.1371/journal.pone.0227690 (PMC6991959; doi:10.1371/journal.pone.0227690)
Supplement: S1 Fig — Agarose gel electrophoresis after PCR with cDNA generated from tilapia ovary (O), testis (T), gill (G) and eye (E). Fragments were around 280 nucleotides in length for gtf3aa and around 130 for gtf3ab. An unspecific amplicon can be observed in the gtf3ab gels that does not mask the specific ovarian amplicon (arrow). Ø = no template control; L = Standard 50 bp (Invitrogen). (DOC) [file pone.0227690.s001.doc]

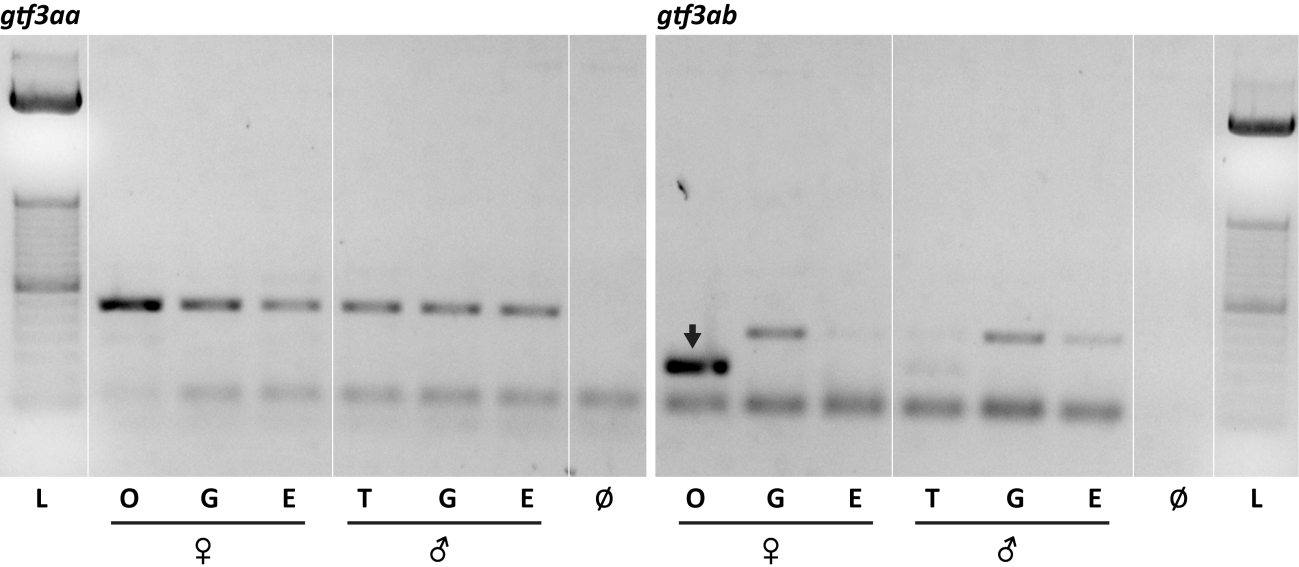


**Figure S1.** *gtf3aa* and *gtf3ab* transcript levels in different tilapia tissues. Agarose gel electrophoresis after conventional PCR with cDNA generated from tilapia ovary (O), testis (T), gill (G) and eye (E). Fragments were around 280 nucleotides in length for *gtf3aa* and around 130 for *gtf3ab*. An unspecific amplicon can be observed in the *gtf3ab* gels that does not mask the specific ovarian amplicon (arrow). Ø=no template control; L= Standard 50 bp (Invitrogen).

|  |  |  | | | | | | | |
| --- | --- | --- | --- | --- | --- | --- | --- | --- | --- |
|  |  |  | | | |  | | | |
|  |  |  |  |  |  |  |  |  |  |
|  |  |  |  |  |  |  |  |  |  |
|  |  |  |  |  |  |  |  |  |  |
|  |  |  |  |  |  |  |  |  |  |
|  |  |  |  |  |  |  |  |  |  |
|  |  |  |  |  |  |  |  |  |  |
|  |  |  |  |  |  |  |  |  |  |
|  |  |  |  |  |  |  |  |  |  |
|  |  |  |  |  |  |  |  |  |  |
|  |  |  |  |  |  |  |  |  |  |
|  |  |  |  |  |  |  |  |  |  |
|  |  |  |  |  |  |  |  |  |  |
|  |  |  |  |  |  |  |  |  |  |
|  |  |  |  |  |  |  |  |  |  |
|  |  |  |  |  |  |  |  |  |  |
|  |  |  |  |  |  |  |  |  |  |
|  |  |  |  |  |  |  |  |  |  |
|  |  |  |  |  |  |  |  |  |  |
|  |  |  |  |  |  |  |  |  |  |
|  |  |  |  |  |  |  |  |  |  |
|  |  |  |  |  |  |  |  |  |  |
|  |  |  |  |  |  |  |  |  |  |
|  |  |  |  |  |  |  |  |  |  |
|  |  |  |  |  |  |  |  |  |  |
|  |  |  |  |  |  |  |  |  |  |
|  |  |  |  |  |  |  |  |  |  |
|  |  |  |  |  |  |  |  |  |  |
|  |  |  |  |  |  |  |  |  |  |
|  |  |  |  |  |  |  |  |  |  |
|  |  |  |  |  |  |  |  |  |  |

|  | |  |  | | | | | | | |  | | | | | | | |
| --- | --- | --- | --- | --- | --- | --- | --- | --- | --- | --- | --- | --- | --- | --- | --- | --- | --- | --- |
|  | |  |  | | | | | | | |  | | | | | | | |
|  | |  |  | |  | |  | | |  |  | |  | |  | |  | |
|  | |  |  | |  | |  | | |  |  | |  | |  | |  | |
|  | |  |  | |  | |  | | |  |  | |  | |  | |  | |
|  | |  |  | |  | |  | | |  |  | |  | |  | |  | |
|  | |  |  | |  | |  | | |  |  | |  | |  | |  | |
|  | |  |  | |  | |  | | |  |  | |  | |  | |  | |
|  | |  |  | |  | |  | | |  |  | |  | |  | |  | |
|  | |  |  | |  | |  | | |  |  | |  | |  | |  | |
|  | |  |  | |  | |  | | |  |  | |  | |  | |  | |
|  | |  |  | |  | |  | | |  |  | |  | |  | |  | |
|  | |  |  | |  | |  | | |  |  | |  | |  | |  | |
|  | |  |  | |  | |  | | |  |  | |  | |  | |  | |
|  | |  |  | |  | |  | | |  |  | |  | |  | |  | |
|  | |  |  | |  | |  | | |  |  | |  | |  | |  | |
|  | |  |  | |  | |  | | |  |  | |  | |  | |  | |
|  | |  |  | |  | |  | | |  |  | |  | |  | |  | |
|  | |  |  | |  | |  | | |  |  | |  | |  | |  | |
|  | |  |  | |  | |  | | |  |  | |  | |  | |  | |
|  | |  |  | |  | |  | | |  |  | |  | |  | |  | |
|  | |  |  | |  | |  | | |  |  | |  | |  | |  | |
|  | |  |  | |  | |  | | |  |  | |  | |  | |  | |
|  | |  |  | |  | |  | | |  |  | |  | |  | |  | |
|  | |  |  | |  | |  | | |  |  | |  | |  | |  | |
|  | |  |  | |  | |  | | |  |  | |  | |  | |  | |
|  | |  |  | |  | |  | | |  |  | |  | |  | |  | |
|  | |  |  | |  | |  | | |  |  | |  | |  | |  | |
|  | |  |  | |  | |  | | |  |  | |  | |  | |  | |
|  | |  |  | |  | |  | | |  |  | |  | |  | |  | |
|  | |  |  | |  | |  | | |  |  | |  | |  | |  | |
|  | |  |  | |  | |  | | |  |  | |  | |  | |  | |
|  |  | |  | | | | | |  | | | | | | | | |  |
|  |  | |  | | | | | |  | | | | | | | | |  |
|  |  | |  |  | |  | |  |  | | |  | |  | |  | |  |
|  |  | |  |  | |  | |  |  | | |  | |  | |  | |  |
|  |  | |  |  | |  | |  |  | | |  | |  | |  | |  |
|  |  | |  |  | |  | |  |  | | |  | |  | |  | |  |
|  |  | |  |  | |  | |  |  | | |  | |  | |  | |  |
|  |  | |  |  | |  | |  |  | | |  | |  | |  | |  |
|  |  | |  |  | |  | |  |  | | |  | |  | |  | |  |
|  |  | |  |  | |  | |  |  | | |  | |  | |  | |  |
|  |  | |  |  | |  | |  |  | | |  | |  | |  | |  |
|  |  | |  |  | |  | |  |  | | |  | |  | |  | |  |
|  |  | |  |  | |  | |  |  | | |  | |  | |  | |  |
|  |  | |  |  | |  | |  |  | | |  | |  | |  | |  |
|  |  | |  |  | |  | |  |  | | |  | |  | |  | |  |
|  |  | |  |  | |  | |  |  | | |  | |  | |  | |  |
|  |  | |  |  | |  | |  |  | | |  | |  | |  | |  |
|  |  | |  |  | |  | |  |  | | |  | |  | |  | |  |
|  |  | |  |  | |  | |  |  | | |  | |  | |  | |  |
|  |  | |  |  | |  | |  |  | | |  | |  | |  | |  |
|  |  | |  |  | |  | |  |  | | |  | |  | |  | |  |
|  |  | |  |  | |  | |  |  | | |  | |  | |  | |  |
|  |  | |  |  | |  | |  |  | | |  | |  | |  | |  |
|  |  | |  |  | |  | |  |  | | |  | |  | |  | |  |
|  |  | |  |  | |  | |  |  | | |  | |  | |  | |  |
|  |  | |  |  | |  | |  |  | | |  | |  | |  | |  |
|  |  | |  |  | |  | |  |  | | |  | |  | |  | |  |
|  |  | |  |  | |  | |  |  | | |  | |  | |  | |  |
|  |  | |  |  | |  | |  |  | | |  | |  | |  | |  |
|  |  | |  |  | |  | |  |  | | |  | |  | |  | |  |
|  |  | |  |  | |  | |  |  | | |  | |  | |  | |  |
|  |  | |  |  | |  | |  |  | | |  | |  | |  | |  |

|  |  |  | | | | | | | |
| --- | --- | --- | --- | --- | --- | --- | --- | --- | --- |
|  |  |  | | | |  | | | |
|  |  |  |  |  |  |  |  |  |  |
|  |  |  |  |  |  |  |  |  |  |
|  |  |  |  |  |  |  |  |  |  |
|  |  |  |  |  |  |  |  |  |  |
|  |  |  |  |  |  |  |  |  |  |
|  |  |  |  |  |  |  |  |  |  |
|  |  |  |  |  |  |  |  |  |  |
|  |  |  |  |  |  |  |  |  |  |
|  |  |  |  |  |  |  |  |  |  |
|  |  |  |  |  |  |  |  |  |  |
|  |  |  |  |  |  |  |  |  |  |
|  |  |  |  |  |  |  |  |  |  |
|  |  |  |  |  |  |  |  |  |  |
|  |  |  |  |  |  |  |  |  |  |
|  |  |  |  |  |  |  |  |  |  |
|  |  |  |  |  |  |  |  |  |  |
|  |  |  |  |  |  |  |  |  |  |
|  |  |  |  |  |  |  |  |  |  |
|  |  |  |  |  |  |  |  |  |  |
|  |  |  |  |  |  |  |  |  |  |
|  |  |  |  |  |  |  |  |  |  |
|  |  |  |  |  |  |  |  |  |  |
|  |  |  |  |  |  |  |  |  |  |
|  |  |  |  |  |  |  |  |  |  |
|  |  |  |  |  |  |  |  |  |  |
|  |  |  |  |  |  |  |  |  |  |
|  |  |  |  |  |  |  |  |  |  |
|  |  |  |  |  |  |  |  |  |  |
|  |  |  |  |  |  |  |  |  |  |
|  |  |  |  |  |  |  |  |  |  |

|  |  |  | | | | | | | |
| --- | --- | --- | --- | --- | --- | --- | --- | --- | --- |
|  |  |  | | | |  | | | |
|  |  |  |  |  |  |  |  |  |  |
|  |  |  |  |  |  |  |  |  |  |
|  |  |  |  |  |  |  |  |  |  |
|  |  |  |  |  |  |  |  |  |  |
|  |  |  |  |  |  |  |  |  |  |
|  |  |  |  |  |  |  |  |  |  |
|  |  |  |  |  |  |  |  |  |  |
|  |  |  |  |  |  |  |  |  |  |
|  |  |  |  |  |  |  |  |  |  |
|  |  |  |  |  |  |  |  |  |  |
|  |  |  |  |  |  |  |  |  |  |
|  |  |  |  |  |  |  |  |  |  |
|  |  |  |  |  |  |  |  |  |  |
|  |  |  |  |  |  |  |  |  |  |
|  |  |  |  |  |  |  |  |  |  |
|  |  |  |  |  |  |  |  |  |  |
|  |  |  |  |  |  |  |  |  |  |
|  |  |  |  |  |  |  |  |  |  |
|  |  |  |  |  |  |  |  |  |  |
|  |  |  |  |  |  |  |  |  |  |
|  |  |  |  |  |  |  |  |  |  |
|  |  |  |  |  |  |  |  |  |  |
|  |  |  |  |  |  |  |  |  |  |
|  |  |  |  |  |  |  |  |  |  |
|  |  |  |  |  |  |  |  |  |  |
|  |  |  |  |  |  |  |  |  |  |
|  |  |  |  |  |  |  |  |  |  |
|  |  |  |  |  |  |  |  |  |  |
|  |  |  |  |  |  |  |  |  |  |

|  |  |  | | | |  | | | |
| --- | --- | --- | --- | --- | --- | --- | --- | --- | --- |
|  |  |  |  |  |  |  |  |  |  |
|  |  |  |  |  |  |  |  |  |  |
|  |  |  |  |  |  |  |  |  |  |
|  |  |  |  |  |  |  |  |  |  |
|  |  |  |  |  |  |  |  |  |  |
|  |  |  |  |  |  |  |  |  |  |
|  |  |  |  |  |  |  |  |  |  |
|  |  |  |  |  |  |  |  |  |  |
|  |  |  |  |  |  |  |  |  |  |
|  |  |  |  |  |  |  |  |  |  |
|  |  |  |  |  |  |  |  |  |  |
|  |  |  |  |  |  |  |  |  |  |
|  |  |  |  |  |  |  |  |  |  |
|  |  |  |  |  |  |  |  |  |  |
|  |  |  |  |  |  |  |  |  |  |
|  |  |  |  |  |  |  |  |  |  |
|  |  |  |  |  |  |  |  |  |  |
|  |  |  |  |  |  |  |  |  |  |
|  |  |  |  |  |  |  |  |  |  |
|  |  |  |  |  |  |  |  |  |  |
|  |  |  |  |  |  |  |  |  |  |
|  |  |  |  |  |  |  |  |  |  |
|  |  |  |  |  |  |  |  |  |  |
|  |  |  |  |  |  |  |  |  |  |
|  |  |  |  |  |  |  |  |  |  |
|  |  |  |  |  |  |  |  |  |  |
|  |  |  |  |  |  |  |  |  |  |
|  |  |  |  |  |  |  |  |  |  |
|  |  |  |  |  |  |  |  |  |  |
|  |  |  |  |  |  |  |  |  |  |

|  |  |  | | | |
| --- | --- | --- | --- | --- | --- |
|  |  |  |  |  |  |
|  |  |  |  |  |  |
|  |  |  |  |  |  |
|  |  |  |  |  |  |
|  |  |  |  |  |  |
|  |  |  |  |  |  |
|  |  |  |  |  |  |
|  |  |  |  |  |  |
|  |  |  |  |  |  |
|  |  |  |  |  |  |
|  |  |  |  |  |  |
|  |  |  |  |  |  |
|  |  |  |  |  |  |
|  |  |  |  |  |  |
|  |  |  |  |  |  |
|  |  |  |  |  |  |
|  |  |  |  |  |  |
|  |  |  |  |  |  |
|  |  |  |  |  |  |
|  |  |  |  |  |  |
|  |  |  |  |  |  |
|  |  |  |  |  |  |
|  |  |  |  |  |  |
|  |  |  |  |  |  |
|  |  |  |  |  |  |
|  |  |  |  |  |  |
|  |  |  |  |  |  |
|  |  |  |  |  |  |
|  |  |  |  |  |  |
|  |  |  |  |  |  |

|  |  |  |  |
| --- | --- | --- | --- |
|  |  |  |  |
|  |  |  |  |
|  |  |  |  |
|  |  |  |  |
|  |  |  |  |
|  |  |  |  |
|  |  |  |  |
|  |  |  |  |
|  |  |  |  |
|  |  |  |  |
|  |  |  |  |
|  |  |  |  |
|  |  |  |  |
|  |  |  |  |
|  |  |  |  |
|  |  |  |  |
|  |  |  |  |
|  |  |  |  |
|  |  |  |  |
|  |  |  |  |
|  |  |  |  |
|  |  |  |  |
|  |  |  |  |
|  |  |  |  |
|  |  |  |  |
|  |  |  |  |
|  |  |  |  |
|  |  |  |  |
|  |  |  |  |
|  |  |  |  |
|  |  |  |  |
|  |  |  |  |
|  |  |  |  |
|  |  |  |  |
|  |  |  |  |
|  |  |  |  |
|  |  |  |  |
|  |  |  |  |
|  |  |  |  |
|  |  |  |  |
|  |  |  |  |
|  |  |  |  |
|  |  |  |  |
|  |  |  |  |
|  |  |  |  |
|  |  |  |  |
|  |  |  |  |
|  |  |  |  |
