# Supplementary material for: Duplication and subfunctionalisation of the general transcription factor IIIA (gtf3a) gene in teleost genomes, with ovarian specific transcription of gtf3ab
Source: PLoS One. 2020 Jan 30;15(1):e0227690. doi: 10.1371/journal.pone.0227690 (PMC6991959; doi:10.1371/journal.pone.0227690)
Supplement: S2 Fig — The experimental groups after 61 days of exposure were: 17β-estradiol (E61), 17α-methyltestosterone (MT61) and ethanol control group which was separated in female and male (ET61_F vs ET61_M) considering gtf3ab transcription levels. Box plots represent the data within the 25th and 75th percentiles, with the median indicated by a line, and top and bottom whiskers indicating the minimum and maximum values (12 individuals per treatment group with 5 individuals in ET61_F and 7 in ET61_M). Different letters indicate significant differences between groups (Kruskal-Wallis, p<0.05). (DOC) [file pone.0227690.s002.doc]

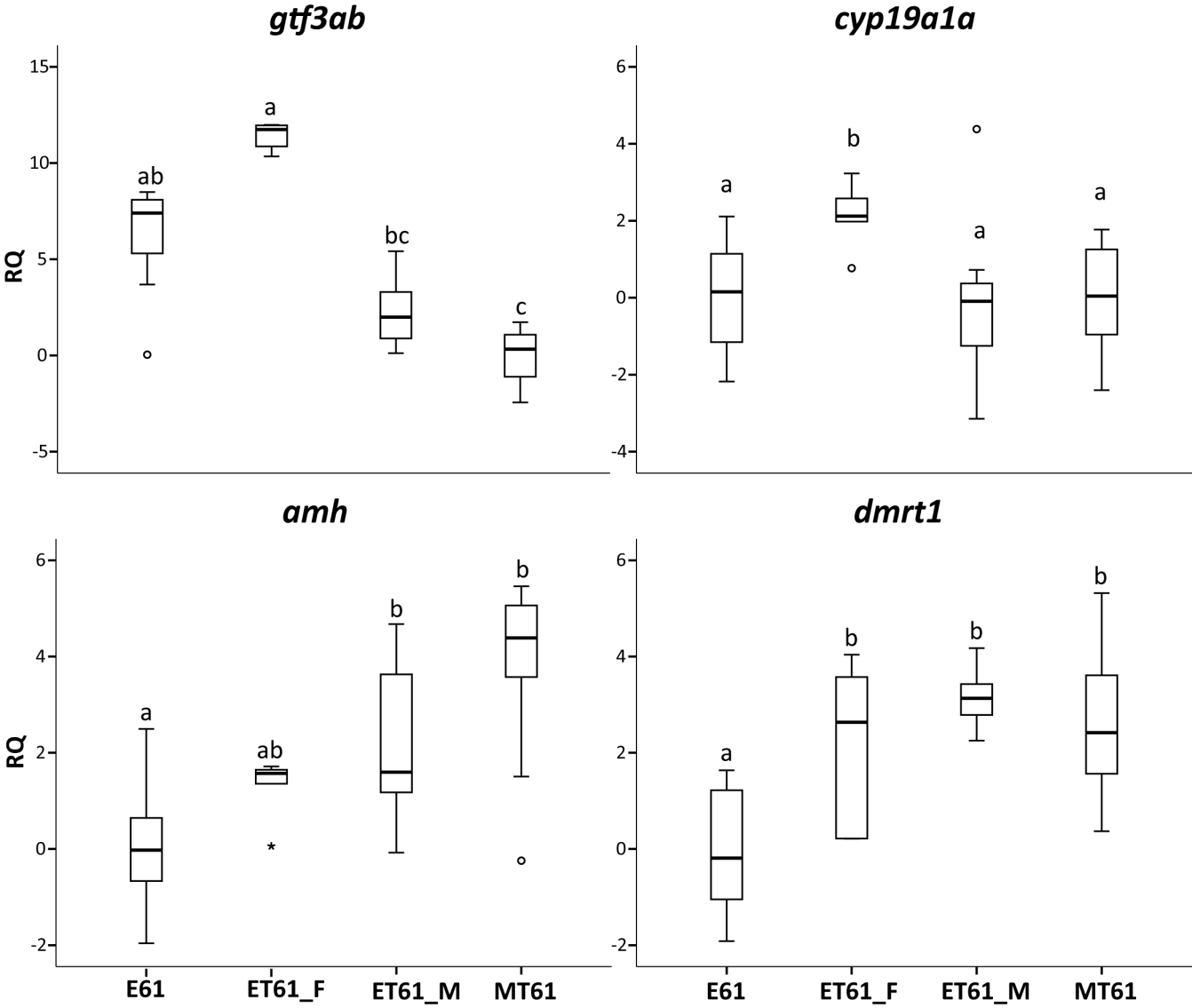


**Figure S2.** Transcript levels of genes related to ovarian (*gtf3ab* and *cyp19a1a*) and testicular (*amh* and *dmrt1*) differentiation in hormone treated zebrafish after 61 days of exposure. The experimental groups after 61 days of exposure were: 17β-estradiol (E61), 17α-methyltestosterone (MT61) and ethanol control group which was separated in female and male (ET61_F vs ET61_M) considering *gtf3ab* transcription levels. Box plots represent the data within the 25th and 75th percentiles, with the median indicated by a line, and top and bottom whiskers indicating the minimum and maximum values (12 individuals per treatment group with 5 individuals in ET61_F and 7 in ET61_M). Different letters indicate significant differences between groups (Kruskal-Wallis, p<0.05).

|  |  |  | | | | | | | |
| --- | --- | --- | --- | --- | --- | --- | --- | --- | --- |
|  |  |  | | | |  | | | |
|  |  |  |  |  |  |  |  |  |  |
|  |  |  |  |  |  |  |  |  |  |
|  |  |  |  |  |  |  |  |  |  |
|  |  |  |  |  |  |  |  |  |  |
|  |  |  |  |  |  |  |  |  |  |
|  |  |  |  |  |  |  |  |  |  |
|  |  |  |  |  |  |  |  |  |  |
|  |  |  |  |  |  |  |  |  |  |
|  |  |  |  |  |  |  |  |  |  |
|  |  |  |  |  |  |  |  |  |  |
|  |  |  |  |  |  |  |  |  |  |
|  |  |  |  |  |  |  |  |  |  |
|  |  |  |  |  |  |  |  |  |  |
|  |  |  |  |  |  |  |  |  |  |
|  |  |  |  |  |  |  |  |  |  |
|  |  |  |  |  |  |  |  |  |  |
|  |  |  |  |  |  |  |  |  |  |
|  |  |  |  |  |  |  |  |  |  |
|  |  |  |  |  |  |  |  |  |  |
|  |  |  |  |  |  |  |  |  |  |
|  |  |  |  |  |  |  |  |  |  |
|  |  |  |  |  |  |  |  |  |  |
|  |  |  |  |  |  |  |  |  |  |
|  |  |  |  |  |  |  |  |  |  |
|  |  |  |  |  |  |  |  |  |  |
|  |  |  |  |  |  |  |  |  |  |
|  |  |  |  |  |  |  |  |  |  |
|  |  |  |  |  |  |  |  |  |  |
|  |  |  |  |  |  |  |  |  |  |
|  |  |  |  |  |  |  |  |  |  |

|  | |  |  | | | | | | | |  | | | | | | | |
| --- | --- | --- | --- | --- | --- | --- | --- | --- | --- | --- | --- | --- | --- | --- | --- | --- | --- | --- |
|  | |  |  | | | | | | | |  | | | | | | | |
|  | |  |  | |  | |  | | |  |  | |  | |  | |  | |
|  | |  |  | |  | |  | | |  |  | |  | |  | |  | |
|  | |  |  | |  | |  | | |  |  | |  | |  | |  | |
|  | |  |  | |  | |  | | |  |  | |  | |  | |  | |
|  | |  |  | |  | |  | | |  |  | |  | |  | |  | |
|  | |  |  | |  | |  | | |  |  | |  | |  | |  | |
|  | |  |  | |  | |  | | |  |  | |  | |  | |  | |
|  | |  |  | |  | |  | | |  |  | |  | |  | |  | |
|  | |  |  | |  | |  | | |  |  | |  | |  | |  | |
|  | |  |  | |  | |  | | |  |  | |  | |  | |  | |
|  | |  |  | |  | |  | | |  |  | |  | |  | |  | |
|  | |  |  | |  | |  | | |  |  | |  | |  | |  | |
|  | |  |  | |  | |  | | |  |  | |  | |  | |  | |
|  | |  |  | |  | |  | | |  |  | |  | |  | |  | |
|  | |  |  | |  | |  | | |  |  | |  | |  | |  | |
|  | |  |  | |  | |  | | |  |  | |  | |  | |  | |
|  | |  |  | |  | |  | | |  |  | |  | |  | |  | |
|  | |  |  | |  | |  | | |  |  | |  | |  | |  | |
|  | |  |  | |  | |  | | |  |  | |  | |  | |  | |
|  | |  |  | |  | |  | | |  |  | |  | |  | |  | |
|  | |  |  | |  | |  | | |  |  | |  | |  | |  | |
|  | |  |  | |  | |  | | |  |  | |  | |  | |  | |
|  | |  |  | |  | |  | | |  |  | |  | |  | |  | |
|  | |  |  | |  | |  | | |  |  | |  | |  | |  | |
|  | |  |  | |  | |  | | |  |  | |  | |  | |  | |
|  | |  |  | |  | |  | | |  |  | |  | |  | |  | |
|  | |  |  | |  | |  | | |  |  | |  | |  | |  | |
|  | |  |  | |  | |  | | |  |  | |  | |  | |  | |
|  | |  |  | |  | |  | | |  |  | |  | |  | |  | |
|  | |  |  | |  | |  | | |  |  | |  | |  | |  | |
|  |  | |  | | | | | |  | | | | | | | | |  |
|  |  | |  | | | | | |  | | | | | | | | |  |
|  |  | |  |  | |  | |  |  | | |  | |  | |  | |  |
|  |  | |  |  | |  | |  |  | | |  | |  | |  | |  |
|  |  | |  |  | |  | |  |  | | |  | |  | |  | |  |
|  |  | |  |  | |  | |  |  | | |  | |  | |  | |  |
|  |  | |  |  | |  | |  |  | | |  | |  | |  | |  |
|  |  | |  |  | |  | |  |  | | |  | |  | |  | |  |
|  |  | |  |  | |  | |  |  | | |  | |  | |  | |  |
|  |  | |  |  | |  | |  |  | | |  | |  | |  | |  |
|  |  | |  |  | |  | |  |  | | |  | |  | |  | |  |
|  |  | |  |  | |  | |  |  | | |  | |  | |  | |  |
|  |  | |  |  | |  | |  |  | | |  | |  | |  | |  |
|  |  | |  |  | |  | |  |  | | |  | |  | |  | |  |
|  |  | |  |  | |  | |  |  | | |  | |  | |  | |  |
|  |  | |  |  | |  | |  |  | | |  | |  | |  | |  |
|  |  | |  |  | |  | |  |  | | |  | |  | |  | |  |
|  |  | |  |  | |  | |  |  | | |  | |  | |  | |  |
|  |  | |  |  | |  | |  |  | | |  | |  | |  | |  |
|  |  | |  |  | |  | |  |  | | |  | |  | |  | |  |
|  |  | |  |  | |  | |  |  | | |  | |  | |  | |  |
|  |  | |  |  | |  | |  |  | | |  | |  | |  | |  |
|  |  | |  |  | |  | |  |  | | |  | |  | |  | |  |
|  |  | |  |  | |  | |  |  | | |  | |  | |  | |  |
|  |  | |  |  | |  | |  |  | | |  | |  | |  | |  |
|  |  | |  |  | |  | |  |  | | |  | |  | |  | |  |
|  |  | |  |  | |  | |  |  | | |  | |  | |  | |  |
|  |  | |  |  | |  | |  |  | | |  | |  | |  | |  |
|  |  | |  |  | |  | |  |  | | |  | |  | |  | |  |
|  |  | |  |  | |  | |  |  | | |  | |  | |  | |  |
|  |  | |  |  | |  | |  |  | | |  | |  | |  | |  |
|  |  | |  |  | |  | |  |  | | |  | |  | |  | |  |

|  |  |  | | | | | | | |
| --- | --- | --- | --- | --- | --- | --- | --- | --- | --- |
|  |  |  | | | |  | | | |
|  |  |  |  |  |  |  |  |  |  |
|  |  |  |  |  |  |  |  |  |  |
|  |  |  |  |  |  |  |  |  |  |
|  |  |  |  |  |  |  |  |  |  |
|  |  |  |  |  |  |  |  |  |  |
|  |  |  |  |  |  |  |  |  |  |
|  |  |  |  |  |  |  |  |  |  |
|  |  |  |  |  |  |  |  |  |  |
|  |  |  |  |  |  |  |  |  |  |
|  |  |  |  |  |  |  |  |  |  |
|  |  |  |  |  |  |  |  |  |  |
|  |  |  |  |  |  |  |  |  |  |
|  |  |  |  |  |  |  |  |  |  |
|  |  |  |  |  |  |  |  |  |  |
|  |  |  |  |  |  |  |  |  |  |
|  |  |  |  |  |  |  |  |  |  |
|  |  |  |  |  |  |  |  |  |  |
|  |  |  |  |  |  |  |  |  |  |
|  |  |  |  |  |  |  |  |  |  |
|  |  |  |  |  |  |  |  |  |  |
|  |  |  |  |  |  |  |  |  |  |
|  |  |  |  |  |  |  |  |  |  |
|  |  |  |  |  |  |  |  |  |  |
|  |  |  |  |  |  |  |  |  |  |
|  |  |  |  |  |  |  |  |  |  |
|  |  |  |  |  |  |  |  |  |  |
|  |  |  |  |  |  |  |  |  |  |
|  |  |  |  |  |  |  |  |  |  |
|  |  |  |  |  |  |  |  |  |  |
|  |  |  |  |  |  |  |  |  |  |

|  |  |  | | | | | | | |
| --- | --- | --- | --- | --- | --- | --- | --- | --- | --- |
|  |  |  | | | |  | | | |
|  |  |  |  |  |  |  |  |  |  |
|  |  |  |  |  |  |  |  |  |  |
|  |  |  |  |  |  |  |  |  |  |
|  |  |  |  |  |  |  |  |  |  |
|  |  |  |  |  |  |  |  |  |  |
|  |  |  |  |  |  |  |  |  |  |
|  |  |  |  |  |  |  |  |  |  |
|  |  |  |  |  |  |  |  |  |  |
|  |  |  |  |  |  |  |  |  |  |
|  |  |  |  |  |  |  |  |  |  |
|  |  |  |  |  |  |  |  |  |  |
|  |  |  |  |  |  |  |  |  |  |
|  |  |  |  |  |  |  |  |  |  |
|  |  |  |  |  |  |  |  |  |  |
|  |  |  |  |  |  |  |  |  |  |
|  |  |  |  |  |  |  |  |  |  |
|  |  |  |  |  |  |  |  |  |  |
|  |  |  |  |  |  |  |  |  |  |
|  |  |  |  |  |  |  |  |  |  |
|  |  |  |  |  |  |  |  |  |  |
|  |  |  |  |  |  |  |  |  |  |
|  |  |  |  |  |  |  |  |  |  |
|  |  |  |  |  |  |  |  |  |  |
|  |  |  |  |  |  |  |  |  |  |
|  |  |  |  |  |  |  |  |  |  |
|  |  |  |  |  |  |  |  |  |  |
|  |  |  |  |  |  |  |  |  |  |
|  |  |  |  |  |  |  |  |  |  |
|  |  |  |  |  |  |  |  |  |  |

|  |  |  | | | |  | | | |
| --- | --- | --- | --- | --- | --- | --- | --- | --- | --- |
|  |  |  |  |  |  |  |  |  |  |
|  |  |  |  |  |  |  |  |  |  |
|  |  |  |  |  |  |  |  |  |  |
|  |  |  |  |  |  |  |  |  |  |
|  |  |  |  |  |  |  |  |  |  |
|  |  |  |  |  |  |  |  |  |  |
|  |  |  |  |  |  |  |  |  |  |
|  |  |  |  |  |  |  |  |  |  |
|  |  |  |  |  |  |  |  |  |  |
|  |  |  |  |  |  |  |  |  |  |
|  |  |  |  |  |  |  |  |  |  |
|  |  |  |  |  |  |  |  |  |  |
|  |  |  |  |  |  |  |  |  |  |
|  |  |  |  |  |  |  |  |  |  |
|  |  |  |  |  |  |  |  |  |  |
|  |  |  |  |  |  |  |  |  |  |
|  |  |  |  |  |  |  |  |  |  |
|  |  |  |  |  |  |  |  |  |  |
|  |  |  |  |  |  |  |  |  |  |
|  |  |  |  |  |  |  |  |  |  |
|  |  |  |  |  |  |  |  |  |  |
|  |  |  |  |  |  |  |  |  |  |
|  |  |  |  |  |  |  |  |  |  |
|  |  |  |  |  |  |  |  |  |  |
|  |  |  |  |  |  |  |  |  |  |
|  |  |  |  |  |  |  |  |  |  |
|  |  |  |  |  |  |  |  |  |  |
|  |  |  |  |  |  |  |  |  |  |
|  |  |  |  |  |  |  |  |  |  |
|  |  |  |  |  |  |  |  |  |  |

|  |  |  | | | |
| --- | --- | --- | --- | --- | --- |
|  |  |  |  |  |  |
|  |  |  |  |  |  |
|  |  |  |  |  |  |
|  |  |  |  |  |  |
|  |  |  |  |  |  |
|  |  |  |  |  |  |
|  |  |  |  |  |  |
|  |  |  |  |  |  |
|  |  |  |  |  |  |
|  |  |  |  |  |  |
|  |  |  |  |  |  |
|  |  |  |  |  |  |
|  |  |  |  |  |  |
|  |  |  |  |  |  |
|  |  |  |  |  |  |
|  |  |  |  |  |  |
|  |  |  |  |  |  |
|  |  |  |  |  |  |
|  |  |  |  |  |  |
|  |  |  |  |  |  |
|  |  |  |  |  |  |
|  |  |  |  |  |  |
|  |  |  |  |  |  |
|  |  |  |  |  |  |
|  |  |  |  |  |  |
|  |  |  |  |  |  |
|  |  |  |  |  |  |
|  |  |  |  |  |  |
|  |  |  |  |  |  |
|  |  |  |  |  |  |

|  |  |  |  |
| --- | --- | --- | --- |
|  |  |  |  |
|  |  |  |  |
|  |  |  |  |
|  |  |  |  |
|  |  |  |  |
|  |  |  |  |
|  |  |  |  |
|  |  |  |  |
|  |  |  |  |
|  |  |  |  |
|  |  |  |  |
|  |  |  |  |
|  |  |  |  |
|  |  |  |  |
|  |  |  |  |
|  |  |  |  |
|  |  |  |  |
|  |  |  |  |
|  |  |  |  |
|  |  |  |  |
|  |  |  |  |
|  |  |  |  |
|  |  |  |  |
|  |  |  |  |
|  |  |  |  |
|  |  |  |  |
|  |  |  |  |
|  |  |  |  |
|  |  |  |  |
|  |  |  |  |
|  |  |  |  |
|  |  |  |  |
|  |  |  |  |
|  |  |  |  |
|  |  |  |  |
|  |  |  |  |
|  |  |  |  |
|  |  |  |  |
|  |  |  |  |
|  |  |  |  |
|  |  |  |  |
|  |  |  |  |
|  |  |  |  |
|  |  |  |  |
|  |  |  |  |
|  |  |  |  |
|  |  |  |  |
|  |  |  |  |
