# Supplementary material for: Duplication and subfunctionalisation of the general transcription factor IIIA (gtf3a) gene in teleost genomes, with ovarian specific transcription of gtf3ab
Source: PLoS One. 2020 Jan 30;15(1):e0227690. doi: 10.1371/journal.pone.0227690 (PMC6991959; doi:10.1371/journal.pone.0227690)
Supplement: S3 Fig — Notice that mtif3 and gtf3aa are always similarly transcribed in all depicted tissues and developmental stages. (DOC) [file pone.0227690.s003.doc]

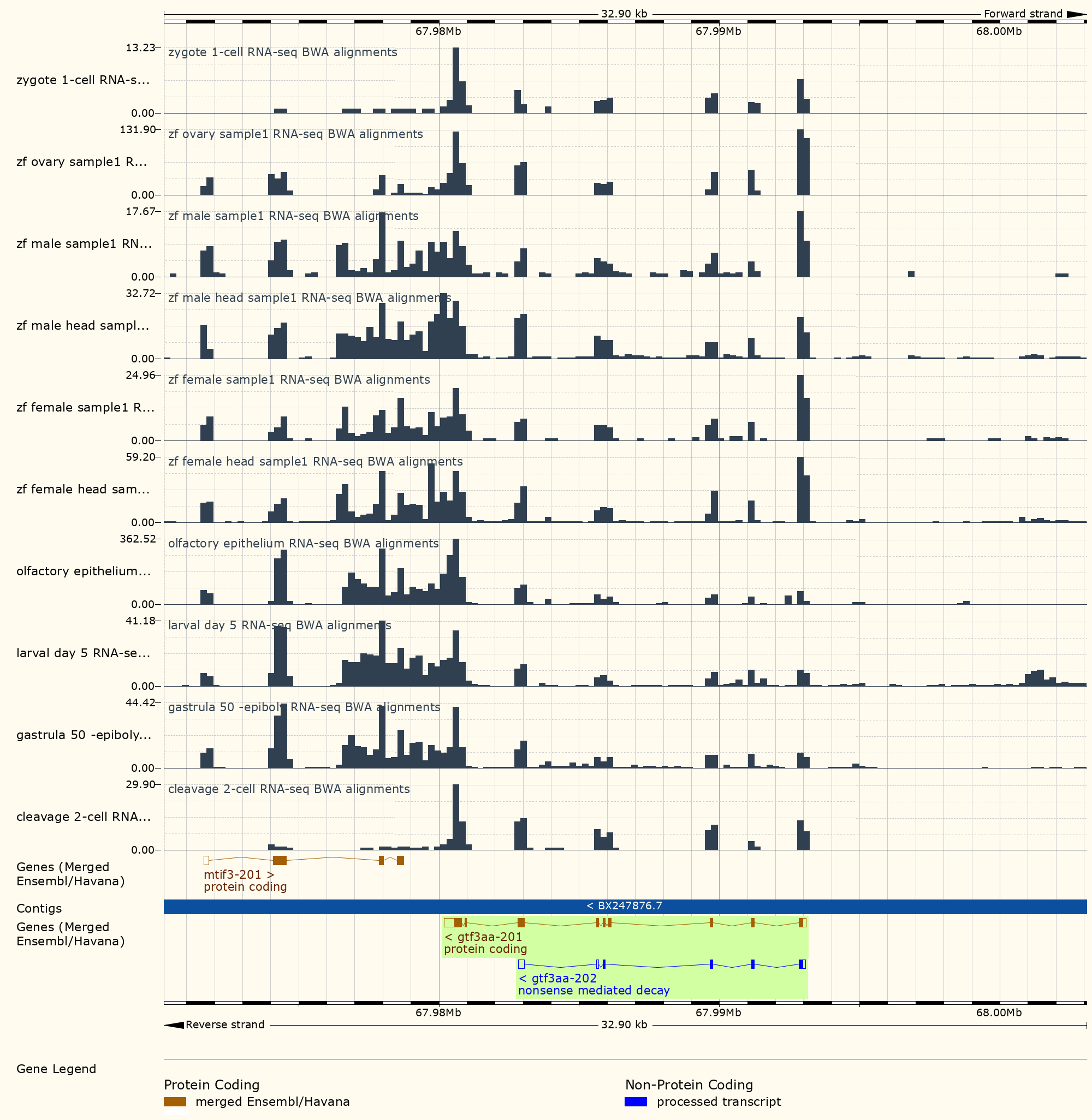
 **Figure S3**. Figure generated in Ensembl with the data obtained from different RNA-seq experiments in zebrafish and that depicts transcript levels in different tissues superimposed on the region of the genome where *gtf3aa* is located. Notice that *mtif3* and *gtf3aa* are always similarly transcribed in all depicted tissues and developmental stages.

|  |  |  | | | | | | | |
| --- | --- | --- | --- | --- | --- | --- | --- | --- | --- |
|  |  |  | | | |  | | | |
|  |  |  |  |  |  |  |  |  |  |
|  |  |  |  |  |  |  |  |  |  |
|  |  |  |  |  |  |  |  |  |  |
|  |  |  |  |  |  |  |  |  |  |
|  |  |  |  |  |  |  |  |  |  |
|  |  |  |  |  |  |  |  |  |  |
|  |  |  |  |  |  |  |  |  |  |
|  |  |  |  |  |  |  |  |  |  |
|  |  |  |  |  |  |  |  |  |  |
|  |  |  |  |  |  |  |  |  |  |
|  |  |  |  |  |  |  |  |  |  |
|  |  |  |  |  |  |  |  |  |  |
|  |  |  |  |  |  |  |  |  |  |
|  |  |  |  |  |  |  |  |  |  |
|  |  |  |  |  |  |  |  |  |  |
|  |  |  |  |  |  |  |  |  |  |
|  |  |  |  |  |  |  |  |  |  |
|  |  |  |  |  |  |  |  |  |  |
|  |  |  |  |  |  |  |  |  |  |
|  |  |  |  |  |  |  |  |  |  |
|  |  |  |  |  |  |  |  |  |  |
|  |  |  |  |  |  |  |  |  |  |
|  |  |  |  |  |  |  |  |  |  |
|  |  |  |  |  |  |  |  |  |  |
|  |  |  |  |  |  |  |  |  |  |
|  |  |  |  |  |  |  |  |  |  |
|  |  |  |  |  |  |  |  |  |  |
|  |  |  |  |  |  |  |  |  |  |
|  |  |  |  |  |  |  |  |  |  |
|  |  |  |  |  |  |  |  |  |  |

|  | |  |  | | | | | | | |  | | | | | | | |
| --- | --- | --- | --- | --- | --- | --- | --- | --- | --- | --- | --- | --- | --- | --- | --- | --- | --- | --- |
|  | |  |  | | | | | | | |  | | | | | | | |
|  | |  |  | |  | |  | | |  |  | |  | |  | |  | |
|  | |  |  | |  | |  | | |  |  | |  | |  | |  | |
|  | |  |  | |  | |  | | |  |  | |  | |  | |  | |
|  | |  |  | |  | |  | | |  |  | |  | |  | |  | |
|  | |  |  | |  | |  | | |  |  | |  | |  | |  | |
|  | |  |  | |  | |  | | |  |  | |  | |  | |  | |
|  | |  |  | |  | |  | | |  |  | |  | |  | |  | |
|  | |  |  | |  | |  | | |  |  | |  | |  | |  | |
|  | |  |  | |  | |  | | |  |  | |  | |  | |  | |
|  | |  |  | |  | |  | | |  |  | |  | |  | |  | |
|  | |  |  | |  | |  | | |  |  | |  | |  | |  | |
|  | |  |  | |  | |  | | |  |  | |  | |  | |  | |
|  | |  |  | |  | |  | | |  |  | |  | |  | |  | |
|  | |  |  | |  | |  | | |  |  | |  | |  | |  | |
|  | |  |  | |  | |  | | |  |  | |  | |  | |  | |
|  | |  |  | |  | |  | | |  |  | |  | |  | |  | |
|  | |  |  | |  | |  | | |  |  | |  | |  | |  | |
|  | |  |  | |  | |  | | |  |  | |  | |  | |  | |
|  | |  |  | |  | |  | | |  |  | |  | |  | |  | |
|  | |  |  | |  | |  | | |  |  | |  | |  | |  | |
|  | |  |  | |  | |  | | |  |  | |  | |  | |  | |
|  | |  |  | |  | |  | | |  |  | |  | |  | |  | |
|  | |  |  | |  | |  | | |  |  | |  | |  | |  | |
|  | |  |  | |  | |  | | |  |  | |  | |  | |  | |
|  | |  |  | |  | |  | | |  |  | |  | |  | |  | |
|  | |  |  | |  | |  | | |  |  | |  | |  | |  | |
|  | |  |  | |  | |  | | |  |  | |  | |  | |  | |
|  | |  |  | |  | |  | | |  |  | |  | |  | |  | |
|  | |  |  | |  | |  | | |  |  | |  | |  | |  | |
|  | |  |  | |  | |  | | |  |  | |  | |  | |  | |
|  |  | |  | | | | | |  | | | | | | | | |  |
|  |  | |  | | | | | |  | | | | | | | | |  |
|  |  | |  |  | |  | |  |  | | |  | |  | |  | |  |
|  |  | |  |  | |  | |  |  | | |  | |  | |  | |  |
|  |  | |  |  | |  | |  |  | | |  | |  | |  | |  |
|  |  | |  |  | |  | |  |  | | |  | |  | |  | |  |
|  |  | |  |  | |  | |  |  | | |  | |  | |  | |  |
|  |  | |  |  | |  | |  |  | | |  | |  | |  | |  |
|  |  | |  |  | |  | |  |  | | |  | |  | |  | |  |
|  |  | |  |  | |  | |  |  | | |  | |  | |  | |  |
|  |  | |  |  | |  | |  |  | | |  | |  | |  | |  |
|  |  | |  |  | |  | |  |  | | |  | |  | |  | |  |
|  |  | |  |  | |  | |  |  | | |  | |  | |  | |  |
|  |  | |  |  | |  | |  |  | | |  | |  | |  | |  |
|  |  | |  |  | |  | |  |  | | |  | |  | |  | |  |
|  |  | |  |  | |  | |  |  | | |  | |  | |  | |  |
|  |  | |  |  | |  | |  |  | | |  | |  | |  | |  |
|  |  | |  |  | |  | |  |  | | |  | |  | |  | |  |
|  |  | |  |  | |  | |  |  | | |  | |  | |  | |  |
|  |  | |  |  | |  | |  |  | | |  | |  | |  | |  |
|  |  | |  |  | |  | |  |  | | |  | |  | |  | |  |
|  |  | |  |  | |  | |  |  | | |  | |  | |  | |  |
|  |  | |  |  | |  | |  |  | | |  | |  | |  | |  |
|  |  | |  |  | |  | |  |  | | |  | |  | |  | |  |
|  |  | |  |  | |  | |  |  | | |  | |  | |  | |  |
|  |  | |  |  | |  | |  |  | | |  | |  | |  | |  |
|  |  | |  |  | |  | |  |  | | |  | |  | |  | |  |
|  |  | |  |  | |  | |  |  | | |  | |  | |  | |  |
|  |  | |  |  | |  | |  |  | | |  | |  | |  | |  |
|  |  | |  |  | |  | |  |  | | |  | |  | |  | |  |
|  |  | |  |  | |  | |  |  | | |  | |  | |  | |  |
|  |  | |  |  | |  | |  |  | | |  | |  | |  | |  |

|  |  |  | | | | | | | |
| --- | --- | --- | --- | --- | --- | --- | --- | --- | --- |
|  |  |  | | | |  | | | |
|  |  |  |  |  |  |  |  |  |  |
|  |  |  |  |  |  |  |  |  |  |
|  |  |  |  |  |  |  |  |  |  |
|  |  |  |  |  |  |  |  |  |  |
|  |  |  |  |  |  |  |  |  |  |
|  |  |  |  |  |  |  |  |  |  |
|  |  |  |  |  |  |  |  |  |  |
|  |  |  |  |  |  |  |  |  |  |
|  |  |  |  |  |  |  |  |  |  |
|  |  |  |  |  |  |  |  |  |  |
|  |  |  |  |  |  |  |  |  |  |
|  |  |  |  |  |  |  |  |  |  |
|  |  |  |  |  |  |  |  |  |  |
|  |  |  |  |  |  |  |  |  |  |
|  |  |  |  |  |  |  |  |  |  |
|  |  |  |  |  |  |  |  |  |  |
|  |  |  |  |  |  |  |  |  |  |
|  |  |  |  |  |  |  |  |  |  |
|  |  |  |  |  |  |  |  |  |  |
|  |  |  |  |  |  |  |  |  |  |
|  |  |  |  |  |  |  |  |  |  |
|  |  |  |  |  |  |  |  |  |  |
|  |  |  |  |  |  |  |  |  |  |
|  |  |  |  |  |  |  |  |  |  |
|  |  |  |  |  |  |  |  |  |  |
|  |  |  |  |  |  |  |  |  |  |
|  |  |  |  |  |  |  |  |  |  |
|  |  |  |  |  |  |  |  |  |  |
|  |  |  |  |  |  |  |  |  |  |
|  |  |  |  |  |  |  |  |  |  |

|  |  |  | | | | | | | |
| --- | --- | --- | --- | --- | --- | --- | --- | --- | --- |
|  |  |  | | | |  | | | |
|  |  |  |  |  |  |  |  |  |  |
|  |  |  |  |  |  |  |  |  |  |
|  |  |  |  |  |  |  |  |  |  |
|  |  |  |  |  |  |  |  |  |  |
|  |  |  |  |  |  |  |  |  |  |
|  |  |  |  |  |  |  |  |  |  |
|  |  |  |  |  |  |  |  |  |  |
|  |  |  |  |  |  |  |  |  |  |
|  |  |  |  |  |  |  |  |  |  |
|  |  |  |  |  |  |  |  |  |  |
|  |  |  |  |  |  |  |  |  |  |
|  |  |  |  |  |  |  |  |  |  |
|  |  |  |  |  |  |  |  |  |  |
|  |  |  |  |  |  |  |  |  |  |
|  |  |  |  |  |  |  |  |  |  |
|  |  |  |  |  |  |  |  |  |  |
|  |  |  |  |  |  |  |  |  |  |
|  |  |  |  |  |  |  |  |  |  |
|  |  |  |  |  |  |  |  |  |  |
|  |  |  |  |  |  |  |  |  |  |
|  |  |  |  |  |  |  |  |  |  |
|  |  |  |  |  |  |  |  |  |  |
|  |  |  |  |  |  |  |  |  |  |
|  |  |  |  |  |  |  |  |  |  |
|  |  |  |  |  |  |  |  |  |  |
|  |  |  |  |  |  |  |  |  |  |
|  |  |  |  |  |  |  |  |  |  |
|  |  |  |  |  |  |  |  |  |  |
|  |  |  |  |  |  |  |  |  |  |

|  |  |  | | | |  | | | |
| --- | --- | --- | --- | --- | --- | --- | --- | --- | --- |
|  |  |  |  |  |  |  |  |  |  |
|  |  |  |  |  |  |  |  |  |  |
|  |  |  |  |  |  |  |  |  |  |
|  |  |  |  |  |  |  |  |  |  |
|  |  |  |  |  |  |  |  |  |  |
|  |  |  |  |  |  |  |  |  |  |
|  |  |  |  |  |  |  |  |  |  |
|  |  |  |  |  |  |  |  |  |  |
|  |  |  |  |  |  |  |  |  |  |
|  |  |  |  |  |  |  |  |  |  |
|  |  |  |  |  |  |  |  |  |  |
|  |  |  |  |  |  |  |  |  |  |
|  |  |  |  |  |  |  |  |  |  |
|  |  |  |  |  |  |  |  |  |  |
|  |  |  |  |  |  |  |  |  |  |
|  |  |  |  |  |  |  |  |  |  |
|  |  |  |  |  |  |  |  |  |  |
|  |  |  |  |  |  |  |  |  |  |
|  |  |  |  |  |  |  |  |  |  |
|  |  |  |  |  |  |  |  |  |  |
|  |  |  |  |  |  |  |  |  |  |
|  |  |  |  |  |  |  |  |  |  |
|  |  |  |  |  |  |  |  |  |  |
|  |  |  |  |  |  |  |  |  |  |
|  |  |  |  |  |  |  |  |  |  |
|  |  |  |  |  |  |  |  |  |  |
|  |  |  |  |  |  |  |  |  |  |
|  |  |  |  |  |  |  |  |  |  |
|  |  |  |  |  |  |  |  |  |  |
|  |  |  |  |  |  |  |  |  |  |

|  |  |  | | | |
| --- | --- | --- | --- | --- | --- |
|  |  |  |  |  |  |
|  |  |  |  |  |  |
|  |  |  |  |  |  |
|  |  |  |  |  |  |
|  |  |  |  |  |  |
|  |  |  |  |  |  |
|  |  |  |  |  |  |
|  |  |  |  |  |  |
|  |  |  |  |  |  |
|  |  |  |  |  |  |
|  |  |  |  |  |  |
|  |  |  |  |  |  |
|  |  |  |  |  |  |
|  |  |  |  |  |  |
|  |  |  |  |  |  |
|  |  |  |  |  |  |
|  |  |  |  |  |  |
|  |  |  |  |  |  |
|  |  |  |  |  |  |
|  |  |  |  |  |  |
|  |  |  |  |  |  |
|  |  |  |  |  |  |
|  |  |  |  |  |  |
|  |  |  |  |  |  |
|  |  |  |  |  |  |
|  |  |  |  |  |  |
|  |  |  |  |  |  |
|  |  |  |  |  |  |
|  |  |  |  |  |  |
|  |  |  |  |  |  |

|  |  |  |  |
| --- | --- | --- | --- |
|  |  |  |  |
|  |  |  |  |
|  |  |  |  |
|  |  |  |  |
|  |  |  |  |
|  |  |  |  |
|  |  |  |  |
|  |  |  |  |
|  |  |  |  |
|  |  |  |  |
|  |  |  |  |
|  |  |  |  |
|  |  |  |  |
|  |  |  |  |
|  |  |  |  |
|  |  |  |  |
|  |  |  |  |
|  |  |  |  |
|  |  |  |  |
|  |  |  |  |
|  |  |  |  |
|  |  |  |  |
|  |  |  |  |
|  |  |  |  |
|  |  |  |  |
|  |  |  |  |
|  |  |  |  |
|  |  |  |  |
|  |  |  |  |
|  |  |  |  |
|  |  |  |  |
|  |  |  |  |
|  |  |  |  |
|  |  |  |  |
|  |  |  |  |
|  |  |  |  |
|  |  |  |  |
|  |  |  |  |
|  |  |  |  |
|  |  |  |  |
|  |  |  |  |
|  |  |  |  |
|  |  |  |  |
|  |  |  |  |
|  |  |  |  |
|  |  |  |  |
|  |  |  |  |
|  |  |  |  |
