# Supplementary figures and images for: Duplication and subfunctionalisation of the general transcription factor IIIA (gtf3a) gene in teleost genomes, with ovarian specific transcription of gtf3ab
Source: PLoS One. 2020 Jan 30;15(1):e0227690. doi: 10.1371/journal.pone.0227690 (PMC6991959; doi:10.1371/journal.pone.0227690)

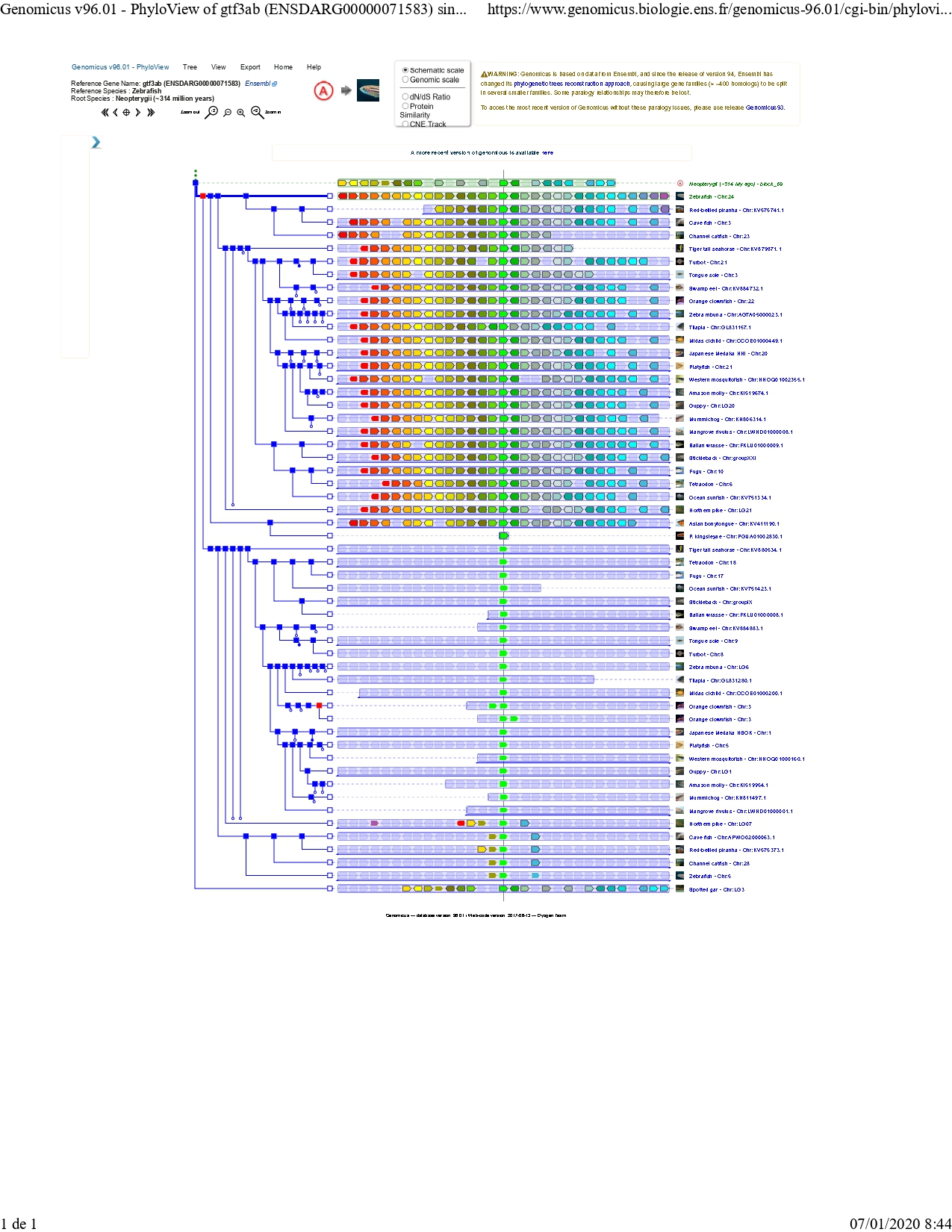


***gtf3ab***


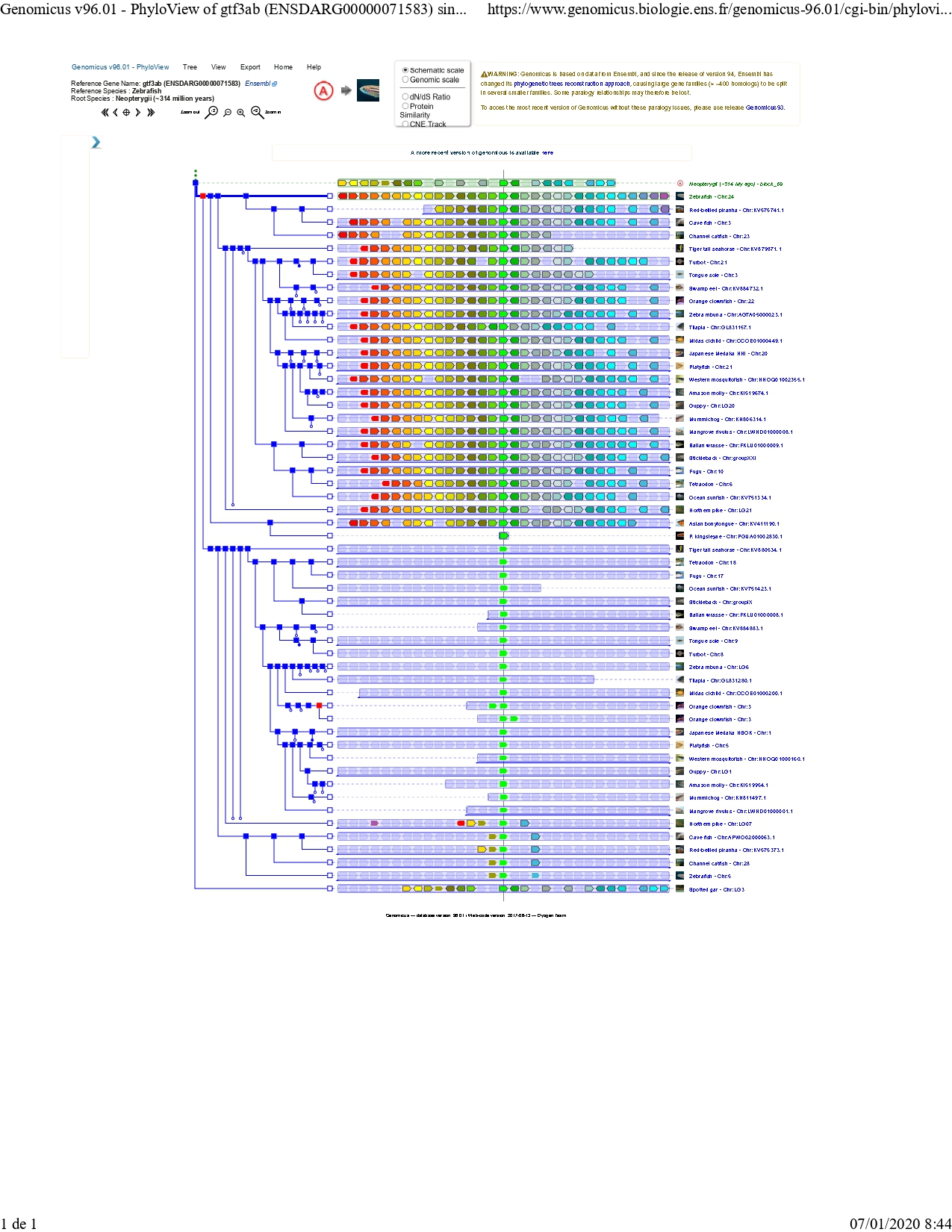


***gtf3a***

***gtf3aa***

Supplement: S5 Fig — (DOCX) [file pone.0227690.s005.docx]
