# Supplementary material for: Duplication and subfunctionalisation of the general transcription factor IIIA (gtf3a) gene in teleost genomes, with ovarian specific transcription of gtf3ab
Source: PLoS One. 2020 Jan 30;15(1):e0227690. doi: 10.1371/journal.pone.0227690 (PMC6991959; doi:10.1371/journal.pone.0227690)
Supplement: S1 Table — (DOC) [file pone.0227690.s006.doc]

**Table S1: Ensembl reference IDs and locations for each of the genes neighbouring *gtf3a* orthologs in animal genomes studied in the synteny analysis.**

|  |  | **Tetrapoda** | | | | | | | |
| --- | --- | --- | --- | --- | --- | --- | --- | --- | --- |
|  |  | **Human (*Homo sapiens*)** | | | | **Frog (*Xenopus tropicalis*)** | | | |
| Gene symbol | Name | ID | Location | Start-End | Strand | ID | Location | Start-End | Strand |
| *spata13* | spermatogenesis associated 13 | ENSG00000182957 | Chr 13 | 23.97-24.30 Mbp | + |  |  |  |  |
| *c1qtnf9* | C1q tumor necrosis factor related protein 9 | ENSG00000240654 | Chr 13 | 24.30-24.32 Mbp | + |  |  |  |  |
| *mtmr6* | myotubularin related protein 6 | ENSG00000139505 | Chr 13 | 25.24-25.28 Mbp | - |  |  |  |  |
| *nup58* | nucleoporin 58KDa | ENSG00000139496 | Chr 13 | 25.30-25.34 Mbp | + |  |  |  |  |
| *atp8a2* | ATPase. aminophospholiìd transporter. class I. type 8A. member 2 | ENSG00000132932 | Chr 13 | 25.37-26.02 Mbp | + |  |  |  |  |
| *shisa2* | shisa family member 2 | ENSG00000180730 | Chr 13 | 26.04-26.05 Mbp | - |  |  |  |  |
| *rnf6* | ring finger protein (C3H2C3 type) 6 | ENSG00000127870 | Chr 13 | 26.13-26.22 Mbp | - |  |  |  |  |
| *cdk8* | cyclin-dependent kinase 8 | ENSG00000132964 | Chr 13 | 26.25-26.40 Mbp | + | ENSXETG00000009787 | Chr: GL173431.1 | 3.15-23.14 kbp | + |
| *wasf3(b)* | was protein family member 3 (3b) | ENSG00000132970 | Chr 13 | 26.55-26.68 Mbp | + | ENSXETG00000032158 | Chr: GL173431.1 | 72.44-105.24 kbp | + |
| *wasf3a* | was protein family member 3a |  |  |  |  |  |  |  |  |
| *grp12* | G-protein-coupled receptor 12 | ENSG00000132975 | Chr 13 | 26.75-26.76 Mbp | - | ENSXETG00000030348 | Chr: GL173431.1 | 127.68-128.66 kbp | - |
| *usp12(a)* | ubiquitin specific peptidase 12 (12a) | ENSG00000152484 | Chr 13 | 27.06-27.17 Mbp | - | (ENSXETG00000009799 | Chr: GL173431.1 | 197.96-211.61kKbp | - |
| *rpl21* | ribosomal protein L21 | ENSG00000122026 | Chr 13 | 27.25-27.26 Mbp | + | ENSXETG00000009802 | Chr: GL173431.1 | 254.09-258.49 kbp | + |
| *rasl11a* | RAS-like family II member A | ENSG00000122035 | Chr 13 | 27.27-27.27 Mbp | + | ENSXETG00000030983 | Chr: GL173431.1 | 262.67-267.33 kbp | + |
| *gtf3a(b)* | general transcription factor IIIA (A. b) | ENSG00000122034 | Chr 13 | 27.42-27.43 Mbp | + | ENSXETG00000032930 | Chr: GL173431.1 | 348.26-358.24 kbp | + |
| *gtf3aa* | general transcription factor IIIAa |  |  |  |  |  |  |  |  |
| *mtlf3* | mitochondrial translational initiation factor 3 | ENSG00000122033 | Chr 13 | 27.43-27.45 Mbp | - | ENSXETG00000031401 | Chr: GL173431.1 | 231.37-237.20 kbp | - |
| *lnx2(a)* | ligand of numb-protein X2 | ENSG00000139517 | Chr 13 | 27.54-27.62 Mbp | - | ENSXETG00000031406 | Chr: GL173431.1 | 392.66-399.80 kbp | - |
| *gsx1* | GS homeobox 1 | ENSG00000169840 | Chr 13 | 27.79-27.79 Mbp | + |  |  |  |  |
| *pdx1* | pancreatic and duodenal homeobox 1 | ENSG00000139515 | Chr 13 | 27.90-27.92 Mbp | + |  |  |  |  |
| *cdx2* | caudal type homeobox 2 | ENSG00000165556 | Chr 13 | 27.96-27.97 Mbp | - |  |  |  |  |
| *urad* | ureidoimidazoline (2-oxo-4-hydroxy-4-carboxy-5) decarboxylase | ENSG00000183463 | Chr 13 | 27.97-27.98 Mbp | - |  |  |  |  |
| *ftl3* | fms-related tyrosine kinase 3 | ENSG00000122025 | Chr 13 | 28.00-28.10 Mbp | - |  |  |  |  |
| *pan3* | PAN2 poly(A) specific ribonuclease subunit | ENSG00000152520 | Chr 13 | 28.13-28.29 Mbp | + |  |  |  |  |
| *ftl1* | fms-related tyrosine kinase 1 | ENSG00000102755 | Chr 13 | 28.30-28.49 Mbp | - |  |  |  |  |
| *slc7a1* | solute carrier family 7 | ENSG00000139514 | Chr 13 | 29.50-29.59 Mbp | - |  |  |  |  |
| *lpar2b* | lysophosphatidic acid receptor 2b |  |  |  |  |  |  |  |  |
| *Tagln3a* | *Transgelin 3a* |  |  |  |  |  |  |  |  |
| *Tagln3b* | *Transgelin 3b* |  |  |  |  |  |  |  |  |

|  | |  | **Sarcopterygi** | | | | | | | | **Actinopterygi** | | | | | | | |
| --- | --- | --- | --- | --- | --- | --- | --- | --- | --- | --- | --- | --- | --- | --- | --- | --- | --- | --- |
|  | |  | **Coelacanth (*Latimeria chalumnae*)** | | | | | | | | **Spotted gar (*Lepisosteus oculatus*)** | | | | | | | |
| Gene symbol | | Name | ID | | Location | | Start-End | | | strand | ID | | Location | | Start-End | | strand | |
| spata13 | | spermatogenesis associated 13 |  | |  | |  | | |  | ENSLOCG00000004477 | | Chr: LG3 | | 5.86-5.90 Mbp | | + | |
| c1qtnf9 | | C1q tumor necrosis factor related protein 9 |  | |  | |  | | |  | ENSLOCG00000004497 | | Chr: LG3 | | 5.90-5.91 Mbp | | + | |
| mtmr6 | | myotubularin related protein 6 |  | |  | |  | | |  | ENSLOCG00000004016 | | Chr: LG3 | | 5.20-5.21 Mbp | | - | |
| nup58 | | nucleoporin 58KDa |  | |  | |  | | |  | ENSLOCG00000004040 | | Chr: LG3 | | 5.22-5.26 Mbp | | + | |
| atp8a2 | | ATPase. aminophospholiìd transporter. class I. type 8A. member 2 |  | |  | |  | | |  | ENSLOCG00000004080 | | Chr: LG3 | | 5.25-5.37 Mbp | | - | |
| shisa2 | | shisa family member 2 |  | |  | |  | | |  | ENSLOCG00000004070 | | Chr: LG3 | | 5.24-5.25 Mbp | | + | |
| rnf6 | | ring finger protein (C3H2C3 type) 6 |  | |  | |  | | |  |  | |  | |  | |  | |
| cdk8 | | cyclin-dependent kinase 8 |  | |  | |  | | |  | ENSLOCG00000004124 | | Chr: LG3 | | 5.40-5.42 Mbp | | + | |
| wasf3(b) | | was protein family member 3 (3b) | ENSLACG00000001570 | | Chr: JH126705.1 | | 22.80-187.07 kbp | | | + |  | |  | |  | |  | |
| wasf3a | | was protein family member 3a |  | |  | |  | | |  | ENSLOCG00000004135 | | Chr: LG3 | | 5.44-5.45 Mbp | | + | |
| grp12 | | G-protein-coupled receptor 12 | ENSLACG00000007730 | | Chr: JH126705.1 | | 294.91-295.91 kbp | | | - | ENSLOCG00000018189 | | Chr: LG3 | | 5.46-5.47 Mbp | | - | |
| usp12(a) | | ubiquitin specific peptidase 12 (12a) | ENSLACG00000011615 | | Chr: JH126705.1 | | 619.84-714.08 kbp | | | - | ENSLOCG00000004146 | | Chr: LG3 | | 5.50-5.51 Mbp | | - | |
| rpl21 | | ribosomal protein L21 | ENSLACG00000012678 | | Chr: JH126705.1 | | 749.90-764.35 kbp | | | + | ENSLOCG00000004171 | | Chr: LG3 | | 5.51-5.51 Mbp | | + | |
| rasl11a | | RAS-like family II member A | ENSLACG00000013026 | | Chr: JH126705.1 | | 799.02-803.03 kbp | | | + | ENSLOCG00000004176 | | Chr: LG3 | | 5.51-5.52 Mbp | | + | |
| gtf3a(b) | | general transcription factor IIIA (A. b) | ENSLACG00000014594 | | Chr: JH126705.1 | | 1.05-1.07 Mbp | | | + |  | |  | |  | |  | |
| gtf3aa | | general transcription factor IIIAa |  | |  | |  | | |  | ENSLOCG00000004190 | | Chr: LG3 | | 5.55-5.56 Mbp | | + | |
| mtlf3 | | mitochondrial translational initiation factor 3 | ENSLACG00000014694 | | Chr: JH126705.1 | | 1.07-1.09 Mbp | | | - | ENSLOCG00000004205 | | Chr: LG3 | | 5.55-5.55 Mbp | | - | |
| lnx2(a) | | ligand of numb-protein X2 | ENSLACG00000015015 | | Chr: JH126705.1 | | 1.15-1.21 Mbp | | | - | ENSLOCG00000004220 | | Chr: LG3 | | 5.57-5.6 Mbp | | - | |
| gsx1 | | GS homeobox 1 | ENSLACG00000016379 | | Chr: JH126705.1 | | 1.55-1.55 Mbp | | | + | ENSLOCG00000004253 | | Chr: LG3 | | 5.64-5.65 Mbp | | + | |
| pdx1 | | pancreatic and duodenal homeobox 1 | ENSLACG00000016610 | | Chr: JH126705.1 | | 1.64-1.67 Mbp | | | + | ENSLOCG00000004260 | | Chr: LG3 | | 5.66-5.67 Mbp | | + | |
| cdx2 | | caudal type homeobox 2 | ENSLACG00000016728 | | Chr: JH126705.1 | | 1.70-1.71 Mbp | | | - | ENSLOCG00000004278 | | Chr: LG3 | | 5.67-5.68 Mbp | | - | |
| urad | | ureidoimidazoline (2-oxo-4-hydroxy-4-carboxy-5) decarboxylase | ENSLACG00000022307 | | Chr: JH126705.1 | | 1.72-1.74 Mbp | | | - | ENSLOCG00000004296 | | Chr: LG3 | | 5.68-5.68 Mbp | | - | |
| ftl3 | | fms-related tyrosine kinase 3 |  | |  | |  | | |  | ENSLOCG00000004310 | | Chr: LG3 | | 5.68-5.70 Mbp | | - | |
| pan3 | | PAN2 poly(A) specific ribonuclease subunit | ENSLACG00000017156 | | Chr: JH126705.1 | | 1.93-207 Mbp | | | + | ENSLOCG00000004336 | | Chr: LG3 | | 5.70-5.75 Mbp | | + | |
| ftl1 | | fms-related tyrosine kinase 1 | ENSLACG00000017415 | | Chr: JH126705.1 | | 1.08-2.22 Mbp | | | - | ENSLOCG00000004379 | | Chr: LG3 | | 5.73-5.78 Mbp | | - | |
| slc7a1 | | solute carrier family 7 |  | |  | |  | | |  |  | |  | |  | |  | |
| lpar2b | | lysophosphatidic acid receptor 2b |  | |  | |  | | |  |  | |  | |  | |  | |
| Tagln3a | | Transgelin 3a |  | |  | |  | | |  | ENSLOCG00000004445 | | Chr: LG3 | | 5,844-5,848 Mbp | | - | |
| Tagln3b | | Transgelin 3b |  | |  | |  | | |  | ENSLOCG00000004445 | | Chr: LG3 | | 5,85-5,86 Mbp | | - | |
|  |  | | **Teleostei > Osteoglosomorph** | | | | | | **Teleostei >clupeocephala>>Euteleotei** | | | | | | | | |  |
|  |  | | **Asian bony tongue *(Scleropages formosu*s)** | | | | | | **Northern pike *(Esox lucius*)** | | | | | | | | |  |
| Gene symbol | Name | | ID | Location | | Start-End | | strand | ID | | | Location | | Start-End | | strand | |  |
| spata13 | spermatogenesis associated 13 | |  |  | |  | |  | ENSELUG00000014704 | | | Chr: GL21 | | 17.437-17.456 Mbp | | + | |  |
| c1qtnf9 | C1q tumor necrosis factor related protein 9 | |  |  | |  | |  | ENSELUG00000014736 | | | Chr: GL21 | | 17.435-17.437 Mbp | | + | |  |
| mtmr6 | myotubularin related protein 6 | |  |  | |  | |  | ENSELUG00000009434 | | | Chr: GL21 | | 19.701-19.713 Mbp | | - | |  |
| nup58 | nucleoporin 58KDa | |  |  | |  | |  | ENSELUG00000009379 | | | Chr: GL21 | | 19714-19.722 Mbp | | + | |  |
| atp8a2 | ATPase. aminophospholiìd transporter. class I. type 8A. member 2 | | ENSSFOG00015016128 | Chr: KV411190.1 | | 6,566-6,593 Mbp | | - | ENSELUG00000013910 | | | Chr: GL21 | | 17.664-17.703 Mbp | | + | |  |
| shisa2 | shisa family member 2 | |  |  | |  | |  |  | | |  | |  | |  | |  |
| rnf6 | ring finger protein (C3H2C3 type) 6 | | ENSSFOG00015016098 | Chr: KV411190.1 | | 6,544-6,549 Mbp | | - | ENSELUG00000013910 | | | Chr: GL21 | | 17.649-17.655 Mbp | | + | |  |
| cdk8 | cyclin-dependent kinase 8 | | ENSSFOG00015016005 | Chr: KV411190.1 | | 6,5321-6,543 Mbp | | - | ENSELUG00000014003 | | | Chr: GL21 | | 17.638-17.649 Mbp | | - | |  |
| wasf3(b) | was protein family member 3 (3b) | | ENSSFOG00015015972 | Chr: KV411190.1 | | 6,522-6,532 Mbp | | - | ENSELUG00000014003 | | | Chr: GL21 | | 17.627-17.638 Mbp | |  | |  |
| wasf3a | was protein family member 3a | |  |  | |  | |  | ENSELUG00000021472 | | | Chr: GL07 | | 7.920-7.925 Mbp | | - | |  |
| grp12 | G-protein-coupled receptor 12 | | ENSSFOG00015015928 | Chr: KV411190.1 | | 6,516-6,517 Mbp | | - |  | | |  | |  | |  | |  |
| usp12(a) | ubiquitin specific peptidase 12 (12a) | |  |  | |  | |  | ENSELUG00000014044 | | | Chr: GL21 | | 17.611-17.617 Mbp | | + | |  |
| rpl21 | ribosomal protein L21 | | ENSSFOG00015015916 | Chr: KV411190.1 | | 6,503-6,507 Mbp | | + | ENSELUG00000014073 | | | Chr: GL21 | | :17.607-17.610 Mbp | | - | |  |
| rasl11a | RAS-like family II member A | | ENSSFOG00015015903 | Chr: KV411190.1 | | 6,494-6,501 Mbp | | - |  | | |  | |  | |  | |  |
| gtf3a(b) | general transcription factor IIIA (A. b) | |  |  | |  | |  | ENSELUG00000014083 | | | Chr: GL21 | | 17.598-17.605 Mbp | | - | |  |
| gtf3aa | general transcription factor IIIAa | | ENSSFOG00015015882 | Chr: KV411190.1 | | 6,490-6,497 Mbp | | + | ENSELUG00000021503 | | | Chr: GL07 | | 7.896-7.906 Mbp | | - | |  |
| mtlf3 | mitochondrial translational initiation factor 3 | |  |  | |  | |  | ENSELUG00000021487 | | | Chr: GL07 | | 7.899-7.903 Mbp | | + | |  |
| lnx2(a) | ligand of numb-protein X2 | |  |  | |  | |  | ENSELUG00000014124 | | | Chr: GL21 | | 17.58-17.59 Mbp | | + | |  |
| gsx1 | GS homeobox 1 | |  |  | |  | |  |  | | | C | |  | |  | |  |
| pdx1 | pancreatic and duodenal homeobox 1 | | ENSSFOG00015015849 | Chr: KV411190.1 | | 6,47-6,48 Mbp | | + | ENSELUG00000014165 | | | Chr: GL21 | | 17..573-17..579 Mbp | |  | |  |
| cdx2 | caudal type homeobox 2 | |  |  | |  | |  |  | | |  | |  | |  | |  |
| urad | ureidoimidazoline (2-oxo-4-hydroxy-4-carboxy-5) decarboxylase | | ENSSFOG00015015860 | Chr: KV411190.1 | | 6,477-6,478 Mbp | | - | ENSELUG00000014158 | | | Chr: GL21 | | 17..574-17..576 Mbp | | + | |  |
| ftl3 | fms-related tyrosine kinase 3 | |  | Chr: KV411190.1 | |  | |  |  | | |  | |  | |  | |  |
| pan3 | PAN2 poly(A) specific ribonuclease subunit | | ENSSFOG00015015792 | Chr: KV411190.1 | | 6,408-6,434 Mbp | | + |  | | |  | |  | |  | |  |
| ftl1 | fms-related tyrosine kinase 1 | | ENSSFOG00015015683 | Chr: KV411190.1 | | 6,408-6,424 Mbp | | + |  | | |  | |  | |  | |  |
| slc7a1 | solute carrier family 7 | |  |  | |  | |  |  | | |  | |  | |  | |  |
| lpar2b | lysophosphatidic acid receptor 2b | |  |  | |  | |  |  | | |  | |  | |  | |  |
| Tagln3a | Transgelin 3a | |  |  | |  | |  | ENSELUG00000021549/  ENSELUG00000014748 | | | Chr: GL07/  Chr: GL21 | | 7.842-7.847/  17,430 - 17,434 | | - | |  |
| Tagln3b | Transgelin 3b | | ENSSFOG00015015538 | Chr:KV411190.1 | | 6.351-6.360 Mbp 9 | |  | ENSELUG00000021523/ENSELUG00000014780 | | | Chr: GL07 / Chr: GL21 | | 7,848 - 7,854 / 17.422-17.431 | | + | |  |

|  |  | **Teleostei > Otophysa** | | | | | | | |
| --- | --- | --- | --- | --- | --- | --- | --- | --- | --- |
|  |  | **Zebrafish (*Danio rerio*)** | | | | **Chanel catfish ( )** | | | |
| Gene symbol | Name | ID | Location | Start-End | strand | ID | Location | Start-End | strand |
| spata13 | spermatogenesis associated 13 | ENSDARG00000062837 | Chr: 24 | 21.71-21.75 Mbp | - |  |  |  |  |
| c1qtnf9 | C1q tumor necrosis factor related protein 9 | ENSDARG00000058318 | Chr: 24 | 21.76-21.76 Mbp | - |  |  |  |  |
| mtmr6 | myotubularin related protein 6 |  |  |  |  |  |  |  |  |
| nup58 | nucleoporin 58KDa |  |  |  |  |  |  |  |  |
| atp8a2 | ATPase. aminophospholiìd transporter. class I. type 8A. member 2 | ENSDARG00000077492 | Chr: 24 | 21.22-21.35 Mbp | - | ENSIPUG00000010193 | Chr. 23 | 4.93-4.98 Mbp | -+ |
| shisa2 | shisa family member 2 | ENSDARG00000088232 | Chr: 24 | 21.20-21.21 Mbp | + | ENSIPUG00000010144 |  | 4,917 -4,923 Mbp | + |
| rnf6 | ring finger protein (C3H2C3 type) 6 | ENSDARG00000077955 | Chr: 24 | 21.21-21.36 Mbp | - | ENSIPUG00000010428 | Chr. 23 | 4,994-5,000 Mbp | - |
| cdk8 | cyclin-dependent kinase 8 | ENSDARG00000016496 | Chr: 24 | 21.36-21.39 Mbp | + | ENSIPUG00000010456 | Chr. 23 | 5,001 -5,015 Mbp | + |
| wasf3(b) | was protein family member 3 (3b) | ENSDARG00000062948 | Chr: 24 | 21.39-21.43 Mbp | + | ENSIPUG00000010595 | Chr. 23 | 5,016-5,034 Mbp | + |
| wasf3a | was protein family member 3a | ENSDARG00000059466 | Chr: 5 | 67.32-67.34 Mbp | - |  |  |  |  |
| grp12 | G-protein-coupled receptor 12 | ENSDARG00000062934 | Chr: 24 | 21.43-21.44 Mbp | - | ENSIPUG00000010616 | Chr. 23 | 5,039-5,040 Mbp |  |
| usp12(a) | ubiquitin specific peptidase 12 (12a) | ENSDARG00000078109 | Chr: 24 | 21.45-21.47 Mbp | - | ENSIPUG00000010631 | Chr. 23 | 5,047-5,058 Mbp | - |
| rpl21 | ribosomal protein L21 | ENSDARG00000010516 | Chr: 24 | 21.47-21.48 Mbp | + | ENSIPUG00000010677 | Chr. 23 | 5,058-5,062 Mbp | + |
| rasl11a | RAS-like family II member A |  |  |  |  | ENSIPUG00000020771 | Chr 28 | 16,755-16,759 Mbp | - |
| gtf3a(b) | general transcription factor IIIA (A. b) | ENSDARG00000071583 | Chr: 24 | 21.49-21.49 Mbp | + | ENSIPUG00000010714 | Chr. 23 | 5,071-5,076 Mbp | + |
| gtf3aa | general transcription factor IIIAa | ENSDARG00000030267 | Chr: 5 | 67.30-67.31 Mbp | - | ENSIPUG00000020752 | Chr. 28 | 17,573-17,584 Mbp | - |
| mtlf3 | mitochondrial translational initiation factor 3 | ENSDARG00000039649 | Chr: 5 | 67.29-67.30 Mbp | + | ENSIPUG00000020758 | Chr. 28 | 17,573-17,580 Mbp | + |
| lnx2(a) | ligand of numb-protein X2 | ENSDARG00000029177 | Chr: 24 | 21.50-21.52 Mbp | - | ENSIPUG00000010762 | Chr. 23 | 5,076-5,094 Mbp | - |
| gsx1 | GS homeobox 1 | ENSDARG00000035735 | Chr: 5 | 67.23-67.23 Mbp | - | ENSIPUG00000010830 | Chr. 23 |  | + |
| pdx1 | pancreatic and duodenal homeobox 1 | ENSDARG00000002779 | Chr: 24 | 21.53-21.54 Mbp | + |  |  |  |  |
| cdx2 | caudal type homeobox 2 |  |  |  |  |  |  |  |  |
| urad | ureidoimidazoline (2-oxo-4-hydroxy-4-carboxy-5) decarboxylase | ENSDARG00000071579 | Chr: 24 | 21.54-21.54 Mbp | - | ENSIPUG00000010836 | Chr. 23 | 5,110-5,114 Mbp | - |
| ftl3 | fms-related tyrosine kinase 3 | ENSDARG00000058503 | Chr: 24 | 21.54-21.56 Mbp | - |  |  |  |  |
| pan3 | PAN2 poly(A) specific ribonuclease subunit | ENSDARG00000031637 | Chr: 24 | 21.57-21.60 Mbp | + |  |  |  |  |
| ftl1 | fms-related tyrosine kinase 1 | ENSDARG00000019371 | Chr: 24 | 21.60-21.66 Mbp | - |  |  |  |  |
| slc7a1 | solute carrier family 7 | ENSDARG00000099111 | Chr: 5 | 67.13-67.15 Mbp | + |  |  |  |  |
| lpar2b | lysophosphatidic acid receptor 2b |  |  |  |  |  |  |  |  |
| Tagln3a | Transgelin 3a | ENSDARG00000079805/ | Chr: 5/24 | 67.869-67.878/ Mbp | - | ENSIPUG00000020719 | Chr. 28 | 17,505 - 17,517 Mbp | - |
| Tagln3b | Transgelin 3b | ENSDARG00000076435/ ENSDARG00000058394 | Chr:5/ 24 | 67,884 - 67,895/  21,917 - 21,934 Mbp | - | ENSIPUG00000020736 | Chr. 28 | 17,523 - 17,531 Mbp | + |

|  |  | **Teleoste> Neoteleostei** | | | | | | | |
| --- | --- | --- | --- | --- | --- | --- | --- | --- | --- |
|  |  | **Tetraodon (*Tetraodon negroviridis*)** | | | | **Stickleback (*Gasterasteus aculeatus*)** | | | |
| Gene symbol | Name | ID | Location | Start-End | Strand | ID | Location | Start-End | strand |
| spata13 | spermatogenesis associated 13 | ENSTNIG00000012702 | Chr: 6 | 3.533-5.3537 Mbp | - | ENSGACG00000003047 | Chr group XXI | 7.497-7.501 Mbp | + |
| c1qtnf9 | C1q tumor necrosis factor related protein 9 | ENSTNIG00000012701 | Chr: 6 | 3.523-3.525 Mbp | - | ENSGACG00000003040 | Chr group XXI | 7.487-7.488 Mbp | + |
| mtmr6 | myotubularin related protein 6 |  |  |  |  |  |  |  |  |
| nup58 | nucleoporin 58KDa |  |  |  |  |  |  |  |  |
| atp8a2 | ATPase. aminophospholiìd transporter. class I. type 8A. member 2 | ENSTNIG00000012718 | Chr: 6 | 3.75-3.76 Mbp | - | ENSGACG00000003172 | Chr group XXI | 7.637-7.667 Mbp | + |
| shisa2 | shisa family member 2 |  |  |  |  | ENSGACG00000003182 | Chr group XXI | 7.671-7.675 Mbp | - |
| rnf6 | ring finger protein (C3H2C3 type) 6 | ENSTNIG00000012717 | Chr: 6 | 3.741-3.744 Mbp | - | ENSGACG00000003167 | Chr group XXI | 7.624-7.626 Mbp | + |
| cdk8 | cyclin-dependent kinase 8 | ENSTNIG00000012716 | Chr: 6 | 3.735-3.739 Mbp | + | ENSGACG00000003151 | Chr group XXI | 7.616-7.622 Mbp | - |
| wasf3(b) | was protein family member 3 (3b) | ENSTNIG00000012715 | Chr: 6 | 3.72-3.73 Mbp | + | ENSGACG00000003147 | Chr group XXI | 7.605-7.613 Mbp | - |
| wasf3a | was protein family member 3a |  |  |  |  |  |  |  |  |
| grp12 | G-protein-coupled receptor 12 | ENSTNIG00000012714 | Chr: 6 | 3.722-3.723 Mbp | + | ENSGACG00000003145 | Chr group XXI | 7.600-7.601 Mbp | + |
| usp12(a) | ubiquitin specific peptidase 12 (12a) | ENSTNIG00000012713 | Chr: 6 | 3.717-3.720 Mbp | - | ENSGACG00000003114 | Chr group XXI | 7.593-7.598 Mbp | + |
| rpl21 | ribosomal protein L21 | ENSTNIG00000012712 | Chr: 6 | 3.715-3.717 Mbp |  | ENSGACG00000003110 | Chr group XXI | 7.589-7.592 Mbp | - |
| rasl11a | RAS-like family II member A |  |  |  |  |  |  |  |  |
| gtf3a(b) | general transcription factor IIIA (A. b) | ENSTNIG00000012711 | Chr: 6 | 3.606-3.608 Mbp | + | ENSGACG00000003107 | Chr group XXI | 7.582-7.584 Mbp | - |
| gtf3aa | general transcription factor IIIAa | ENSTNIG00000006308 | Chr: 18 | 10.81-10.82 Mbp | + | ENSGACG00000019821 | Chr group IX | 19.645-19.646 Mbp | - |
| mtlf3 | mitochondrial translational initiation factor 3 |  |  |  |  |  |  |  |  |
| lnx2(a) | ligand of numb-protein X2 | ENSTNIG00000012710 | Chr: 6 | 3.59-3.60 Mbp | - | ENSGACG00000003102 | Chr group XXI | 7.575-7.582 Mbp | + |
| gsx1 | GS homeobox 1 |  |  |  |  |  |  |  |  |
| pdx1 | pancreatic and duodenal homeobox 1 | ENSTNIG00000012708 | Chr: 6 | 3.593-3.596 Mbp | + | ENSGACG00000003099 | Chr group XXI | 7.565-7.567 Mbp | - |
| cdx2 | caudal type homeobox 2 |  |  |  |  |  |  |  |  |
| urad | ureidoimidazoline (2-oxo-4-hydroxy-4-carboxy-5) decarboxylase | ENSTNIG00000012707 | Chr: 6 | 6.592-3.593 Mbp | - | ENSGACG00000003095 | Chr group XXI | 7.564-7.564 Mbp | + |
| ftl3 | fms-related tyrosine kinase 3 | ENSTNIG00000012706 | Chr: 6 | 3.589-3.591 Mbp |  |  |  |  |  |
| pan3 | PAN2 poly(A) specific ribonuclease subunit | ENSTNIG00000012705 | Chr: 6 | 3.579-3.584 Mbp | + | ENSGACG00000003081 | Chr group XXI | 7.545-7.552 Mbp | - |
| ftl1 | fms-related tyrosine kinase 1 | ENSTNIG00000012704 | Chr: 6 | 3.563-3.569 Mbp | - | ENSGACG00000003066 | Chr group XXI | 7.519-7.540 Mbp | + |
| slc7a1 | solute carrier family 7 |  |  |  |  |  |  |  |  |
| Tagln3a | Transgelin 3a | ENSTNIG00000012700 | Chr: 6 | 3.522 – 3.522 Mbp | - | ENSGACG00000003016 | Chr group XXI | 7,478 - 7,481 Mbp | - |
| Tagln3b | Transgelin 3b | ENSTNIG00000012699 | Chr:6 | 3.517 – 3.520 Mbp | - | ENSGACG00000003004 |  | 7,474 - 7,47 Mbp | - |

|  |  | **Amazon molly (*Poelicia formosa*)** | | | | **Tilapia (*Oreochromis niloticus*)** | | | |
| --- | --- | --- | --- | --- | --- | --- | --- | --- | --- |
| Gene symbol | Name | ID | Location | Start-End | strand | ID | Location | Start-End | strand |
| spata13 | spermatogenesis associated 13 | ENSPFOG00000006292 | Chr:KI519674.1 | 560.80-584.84 kbp | + | ENSONIG00000014168 | Chr:GL831157.1 | 597.20-603.35 kbp | - |
| c1qtnf9 | C1q tumor necrosis factor related protein 9 | ENSPFOG00000006247 | Chr:KI519674.1 | 556.36-558.47 kbp | + | ENSONIG00000014169 | Chr:GL831157.1 | 613.33-314.65 kbp | - |
| mtmr6 | myotubularin related protein 6 |  |  |  |  |  |  |  |  |
| nup58 | nucleoporin 58KDa |  |  |  |  |  |  |  |  |
| atp8a2 | ATPase. aminophospholiìd transporter. class I. type 8A. member 2 |  |  |  |  | ENSONIG00000014106 | Chr:GL831157.1 | 324.32-372.98 kbp | - |
| shisa2 | shisa family member 2 | ENSPFOG00000009024 | Chr:KI519674.1 | 855.05-863.58 kbp | - | ENSONIG00000014104 | Chr:GL831157.1 | 315.43-320.98 kbp | + |
| rnf6 | ring finger protein (C3H2C3 type) 6 | ENSPFOG00000008161 | Chr:KI519674.1 | 780.53-790.59 kbp | + | ENSONIG00000014121 | Chr:GL831157.1 | 373.19-382.70 kbp | - |
| cdk8 | cyclin-dependent kinase 8 | ENSPFOG00000008071 | Chr:KI519674.1 | 770.11-781.14 kbp | - | ENSONIG00000014123 | Chr:GL831157.1 | 383.13-391.71 kbp | + |
| wasf3(b) | was protein family member 3 (3b) | ENSPFOG00000008013 | Chr:KI519674.1 | 753.16-767.98 kbp | - | ENSONIG00000014127 | Chr:GL831157.1 | 394.30-405.01 kbp | + |
| wasf3a | was protein family member 3a |  |  |  |  |  |  |  |  |
| grp12 | G-protein-coupled receptor 12 | ENSPFOG00000020724 | Chr:KI519674.1 | 746.45-747.47 kbp | + | ENSONIG00000020418 | Chr:GL831157.1 | 411.48-412.50 kbp | - |
| usp12(a) | ubiquitin specific peptidase 12 (12a) | ENSPFOG00000007941 | Chr:KI519674.1 | 732.08-741.16 kbp | + | ENSONIG00000014128 | Chr:GL831157.1 | 415.14-421.51 kbp | - |
| rpl21 | ribosomal protein L21 | ENSPFOG00000007900 | Chr:KI519674.1 | 728.87-731.79 kbp | - | ENSONIG00000014130 | Chr:GL831157.1 | 423.26-424.80 kbp | + |
| rasl11a | RAS-like family II member A |  |  |  |  |  |  |  |  |
| gtf3a(b) | general transcription factor IIIA (A. b) | ENSPFOG00000007819 | Chr:KI519674.1 | 722.36-724.21 kbp | - | ENSONIG00000014134 | Chr:GL831157.1 | 431.25-434.19 kbp | + |
| gtf3aa | general transcription factor IIIAa | ENSPFOG00000020753 | Chr:KI519954.1 | 49.41-50.40 kbp | + | ENSONIG00000020701 | Chr:GL831280.1 | 1.625-1.626 Mbp | + |
| mtlf3 | mitochondrial translational initiation factor 3 |  |  |  |  |  |  |  |  |
| lnx2(a) | ligand of numb-protein X2 | ENSPFOG00000007692 | Chr:KI519674.1 | 706.28-722.27 kbp | + | ENSONIG00000014132 | Chr:GL831157.1 | 431.24-443.79 kbp | - |
| gsx1 | GS homeobox 1 |  |  |  |  |  |  |  |  |
| pdx1 | pancreatic and duodenal homeobox 1 | ENSPFOG00000007668 | Chr:KI519674.1 | 697.49-699.44 kbp | - | ENSONIG00000014137 | Chr:GL831157.1 | 452.60-455.36 kbp | + |
| cdx2 | caudal type homeobox 2 |  |  |  |  |  |  |  |  |
| urad | ureidoimidazoline (2-oxo-4-hydroxy-4-carboxy-5) decarboxylase | ENSPFOG00000007646 | Chr:KI519674.1 | 696.28-698.15 kbp | + | ENSONIG00000014138 | Chr:GL831157.1 | 455.98-456.64 kbp | - |
| ftl3 | fms-related tyrosine kinase 3 | ENSPFOG00000023223 | Chr:KI519674.1 | 669.52-679.37 kbp | + |  |  |  |  |
| pan3 | PAN2 poly(A) specific ribonuclease subunit | ENSPFOG00000007327 | Chr:KI519674.1 | 645.53-665.72 kbp | - | ENSONIG00000014139 | Chr:GL831157.1 | 511.48-531.75 kbp | + |
| ftl1 | fms-related tyrosine kinase 1 | ENSPFOG00000006735 | Chr:KI519674.1 | 607.81-643.79 kbp | + | ENSONIG00000014141 | Chr:GL831157.1 | 536.33-574.03 kbp | - |
| slc7a1 | solute carrier family 7 |  |  |  |  |  |  |  |  |
| lpar2b | lysophosphatidic acid receptor 2b | ENSPFOG00000008943 | Chr:KI519954.1 | 370.72-394.85 kbp | - |  |  |  |  |
| Tagln3a | Transgelin 3a | ENSPFOG00000006186 | Chr:KI519954.1 | 549.102 – 554.673 kbp | - | ENSONIG00000014172 | Chr:GL831157.1 | 616.635 – 618.242 kbp | - |
| Tagln3b | Transgelin 3b | ENSPFOG00000006141 | Chr:KI519954.1 | 542.085 – 546.397 kbp | - | ENSONIG00000014173 | Chr:GL831157.1 | 620.729 – 624.292 kbp | - |

|  |  | **Atlantic cod (*Gadus morhua*)** | | | |
| --- | --- | --- | --- | --- | --- |
| Gene symbol | Name | ID | Location | Start-End | strand |
| spata13 | spermatogenesis associated 13 | ENSGMOG00000015009 | scaffold_733 | 39.91-53.96 kbp | + |
| c1qtnf9 | C1q tumor necrosis factor related protein 9 | ENSGMOG00000014997 | scaffold_733 | 29.83-31.15 kbp | + |
| mtmr6 | myotubularin related protein 6 |  |  |  |  |
| nup58 | nucleoporin 58KDa |  |  |  |  |
| atp8a2 | ATPase. aminophospholiìd transporter. class I. type 8A. member 2 | ENSGMOG00000015425 | scaffold_733 | 33573-39.228 kbp | + |
| shisa2 | shisa family member 2 | ENSGMOG00000015491 | scaffold_733 | 393.51-402.46 kbp | - |
| rnf6 | ring finger protein (C3H2C3 type) 6 | ENSGMOG00000015404 | scaffold_733 | 318.62-321.22 kbp | + |
| cdk8 | cyclin-dependent kinase 8 | ENSGMOG00000015383 | scaffold_733 | 308.24-316.28 kbp | - |
| wasf3(b) | was protein family member 3 (3b) | ENSGMOG00000015343 | scaffold_733 | 287.94-297.99 kbp | - |
| wasf3a | was protein family member 3a |  |  |  |  |
| grp12 | G-protein-coupled receptor 12 |  |  |  |  |
| usp12(a) | ubiquitin specific peptidase 12 (12a) | ENSGMOG00000015314 | scaffold_733 | 274.64-278.79 kbp | + |
| rpl21 | ribosomal protein L21 | ENSGMOG00000015298 | scaffold_733 | 270.81-273.29 kbp | - |
| rasl11a | RAS-like family II member A |  |  |  |  |
| gtf3a(b) | general transcription factor IIIA (A. b) | ENSGMOG00000015268 | scaffold_733 | 263.73-266.18 kbp | - |
| gtf3aa | general transcription factor IIIAa | ENSGMOG00000010259 | scaffold_4115 | 7.92-8.96 kbp | - |
| mtlf3 | mitochondrial translational initiation factor 3 |  |  |  |  |
| lnx2(a) | ligand of numb-protein X2 | ENSGMOG00000015243 | scaffold_733 | 252.99-263.22 kbp | + |
| gsx1 | GS homeobox 1 |  |  |  |  |
| pdx1 | pancreatic and duodenal homeobox 1 | ENSGMOG00000015225 | scaffold_733 | 240.37-242.89 kbp | - |
| cdx2 | caudal type homeobox 2 |  |  |  |  |
| urad | ureidoimidazoline (2-oxo-4-hydroxy-4-carboxy-5) decarboxylase | ENSGMOG00000015217 | scaffold_733 | 237.56-239.90 kbp | + |
| ftl3 | fms-related tyrosine kinase 3 |  |  |  |  |
| pan3 | PAN2 poly(A) specific ribonuclease subunit | ENSGMOG00000015169 | scaffold_733 | 184.47-213.98 kbp | - |
| ftl1 | fms-related tyrosine kinase 1 | ENSGMOG00000015113 | scaffold_733 | 130.02-178.18 kbp | + |
| slc7a1 | solute carrier family 7 |  |  |  |  |
| lpar2b | lysophosphatidic acid receptor 2b |  |  |  |  |
| Tagln3a | Transgelin 3a |  |  |  |  |
| Tagln3b | Transgelin 3b | ENSGMOG00000014986 | scaffold_733 | 19.990 – 23.556 kbp | - |

|  |  |  |  |
| --- | --- | --- | --- |
|  |  |  |  |
|  |  |  |  |
|  |  |  |  |
|  |  |  |  |
|  |  |  |  |
|  |  |  |  |
|  |  |  |  |
|  |  |  |  |
|  |  |  |  |
|  |  |  |  |
|  |  |  |  |
|  |  |  |  |
|  |  |  |  |
|  |  |  |  |
|  |  |  |  |
|  |  |  |  |
|  |  |  |  |
|  |  |  |  |
|  |  |  |  |
|  |  |  |  |
|  |  |  |  |
|  |  |  |  |
|  |  |  |  |
|  |  |  |  |
|  |  |  |  |
|  |  |  |  |
|  |  |  |  |
|  |  |  |  |
|  |  |  |  |
|  |  |  |  |
|  |  |  |  |
|  |  |  |  |
|  |  |  |  |
|  |  |  |  |
|  |  |  |  |
|  |  |  |  |
|  |  |  |  |
|  |  |  |  |
|  |  |  |  |
|  |  |  |  |
|  |  |  |  |
|  |  |  |  |
|  |  |  |  |
|  |  |  |  |
|  |  |  |  |
|  |  |  |  |
|  |  |  |  |
|  |  |  |  |
