# Supplementary material for: Duplication and subfunctionalisation of the general transcription factor IIIA (gtf3a) gene in teleost genomes, with ovarian specific transcription of gtf3ab
Source: PLoS One. 2020 Jan 30;15(1):e0227690. doi: 10.1371/journal.pone.0227690 (PMC6991959; doi:10.1371/journal.pone.0227690)
Supplement: S3 Table — The sequences presented here were the ones used to produce the phylogenetic tree in S1 Fig. Sequence underlined in yellow show the conserved initial sequence in all the oocyte specific Gtf3ab proteins of fish and the Xenopus oocytic protein. The sequence in blue shows the last of the C2H2 Zn finger domains of all the Gtf3as. In green the conserved transcription activation KRSLAS domain (KRSLAShLsGYPPK), necessary for transcriptional activation of 5S rRNA is shown. In teleostean proteins this is only found in Gtf3aa-s. (DOC) [file pone.0227690.s008.doc]

| ***rtd*** |  |  | | | | | | | |
| --- | --- | --- | --- | --- | --- | --- | --- | --- | --- |
|  |  |  | | | |  | | | |
|  |  |  |  |  |  |  |  |  |  |
|  |  |  |  |  |  |  |  |  |  |
|  |  |  |  |  |  |  |  |  |  |
|  |  |  |  |  |  |  |  |  |  |
|  |  |  |  |  |  |  |  |  |  |
|  |  |  |  |  |  |  |  |  |  |
|  |  |  |  |  |  |  |  |  |  |
|  |  |  |  |  |  |  |  |  |  |
|  |  |  |  |  |  |  |  |  |  |
|  |  |  |  |  |  |  |  |  |  |
|  |  |  |  |  |  |  |  |  |  |
|  |  |  |  |  |  |  |  |  |  |
|  |  |  |  |  |  |  |  |  |  |
|  |  |  |  |  |  |  |  |  |  |
|  |  |  |  |  |  |  |  |  |  |
|  |  |  |  |  |  |  |  |  |  |
|  |  |  |  |  |  |  |  |  |  |
|  |  |  |  |  |  |  |  |  |  |
|  |  |  |  |  |  |  |  |  |  |
|  |  |  |  |  |  |  |  |  |  |
|  |  |  |  |  |  |  |  |  |  |
|  |  |  |  |  |  |  |  |  |  |
|  |  |  |  |  |  |  |  |  |  |
|  |  |  |  |  |  |  |  |  |  |
|  |  |  |  |  |  |  |  |  |  |
|  |  |  |  |  |  |  |  |  |  |
|  |  |  |  |  |  |  |  |  |  |
|  |  |  |  |  |  |  |  |  |  |
|  |  |  |  |  |  |  |  |  |  |
|  |  |  |  |  |  |  |  |  |  |

|  | |  |  | | | | | | | |  | | | | | | | |
| --- | --- | --- | --- | --- | --- | --- | --- | --- | --- | --- | --- | --- | --- | --- | --- | --- | --- | --- |
|  | |  |  | | | | | | | |  | | | | | | | |
|  | |  |  | |  | |  | | |  |  | |  | |  | |  | |
|  | |  |  | |  | |  | | |  |  | |  | |  | |  | |
|  | |  |  | |  | |  | | |  |  | |  | |  | |  | |
|  | |  |  | |  | |  | | |  |  | |  | |  | |  | |
|  | |  |  | |  | |  | | |  |  | |  | |  | |  | |
|  | |  |  | |  | |  | | |  |  | |  | |  | |  | |
|  | |  |  | |  | |  | | |  |  | |  | |  | |  | |
|  | |  |  | |  | |  | | |  |  | |  | |  | |  | |
|  | |  |  | |  | |  | | |  |  | |  | |  | |  | |
|  | |  |  | |  | |  | | |  |  | |  | |  | |  | |
|  | |  |  | |  | |  | | |  |  | |  | |  | |  | |
|  | |  |  | |  | |  | | |  |  | |  | |  | |  | |
|  | |  |  | |  | |  | | |  |  | |  | |  | |  | |
|  | |  |  | |  | |  | | |  |  | |  | |  | |  | |
|  | |  |  | |  | |  | | |  |  | |  | |  | |  | |
|  | |  |  | |  | |  | | |  |  | |  | |  | |  | |
|  | |  |  | |  | |  | | |  |  | |  | |  | |  | |
|  | |  |  | |  | |  | | |  |  | |  | |  | |  | |
|  | |  |  | |  | |  | | |  |  | |  | |  | |  | |
|  | |  |  | |  | |  | | |  |  | |  | |  | |  | |
|  | |  |  | |  | |  | | |  |  | |  | |  | |  | |
|  | |  |  | |  | |  | | |  |  | |  | |  | |  | |
|  | |  |  | |  | |  | | |  |  | |  | |  | |  | |
|  | |  |  | |  | |  | | |  |  | |  | |  | |  | |
|  | |  |  | |  | |  | | |  |  | |  | |  | |  | |
|  | |  |  | |  | |  | | |  |  | |  | |  | |  | |
|  | |  |  | |  | |  | | |  |  | |  | |  | |  | |
|  | |  |  | |  | |  | | |  |  | |  | |  | |  | |
|  | |  |  | |  | |  | | |  |  | |  | |  | |  | |
|  | |  |  | |  | |  | | |  |  | |  | |  | |  | |
|  |  | |  | | | | | |  | | | | | | | | |  |
|  |  | |  | | | | | |  | | | | | | | | |  |
|  |  | |  |  | |  | |  |  | | |  | |  | |  | |  |
|  |  | |  |  | |  | |  |  | | |  | |  | |  | |  |
|  |  | |  |  | |  | |  |  | | |  | |  | |  | |  |
|  |  | |  |  | |  | |  |  | | |  | |  | |  | |  |
|  |  | |  |  | |  | |  |  | | |  | |  | |  | |  |
|  |  | |  |  | |  | |  |  | | |  | |  | |  | |  |
|  |  | |  |  | |  | |  |  | | |  | |  | |  | |  |
|  |  | |  |  | |  | |  |  | | |  | |  | |  | |  |
|  |  | |  |  | |  | |  |  | | |  | |  | |  | |  |
|  |  | |  |  | |  | |  |  | | |  | |  | |  | |  |
|  |  | |  |  | |  | |  |  | | |  | |  | |  | |  |
|  |  | |  |  | |  | |  |  | | |  | |  | |  | |  |
|  |  | |  |  | |  | |  |  | | |  | |  | |  | |  |
|  |  | |  |  | |  | |  |  | | |  | |  | |  | |  |
|  |  | |  |  | |  | |  |  | | |  | |  | |  | |  |
|  |  | |  |  | |  | |  |  | | |  | |  | |  | |  |
|  |  | |  |  | |  | |  |  | | |  | |  | |  | |  |
|  |  | |  |  | |  | |  |  | | |  | |  | |  | |  |
|  |  | |  |  | |  | |  |  | | |  | |  | |  | |  |
|  |  | |  |  | |  | |  |  | | |  | |  | |  | |  |
|  |  | |  |  | |  | |  |  | | |  | |  | |  | |  |
|  |  | |  |  | |  | |  |  | | |  | |  | |  | |  |
|  |  | |  |  | |  | |  |  | | |  | |  | |  | |  |
|  |  | |  |  | |  | |  |  | | |  | |  | |  | |  |
|  |  | |  |  | |  | |  |  | | |  | |  | |  | |  |
|  |  | |  |  | |  | |  |  | | |  | |  | |  | |  |
|  |  | |  |  | |  | |  |  | | |  | |  | |  | |  |
|  |  | |  |  | |  | |  |  | | |  | |  | |  | |  |
|  |  | |  |  | |  | |  |  | | |  | |  | |  | |  |
|  |  | |  |  | |  | |  |  | | |  | |  | |  | |  |

|  |  |  | | | | | | | |
| --- | --- | --- | --- | --- | --- | --- | --- | --- | --- |
|  |  |  | | | |  | | | |
|  |  |  |  |  |  |  |  |  |  |
|  |  |  |  |  |  |  |  |  |  |
|  |  |  |  |  |  |  |  |  |  |
|  |  |  |  |  |  |  |  |  |  |
|  |  |  |  |  |  |  |  |  |  |
|  |  |  |  |  |  |  |  |  |  |
|  |  |  |  |  |  |  |  |  |  |
|  |  |  |  |  |  |  |  |  |  |
|  |  |  |  |  |  |  |  |  |  |
|  |  |  |  |  |  |  |  |  |  |
|  |  |  |  |  |  |  |  |  |  |
|  |  |  |  |  |  |  |  |  |  |
|  |  |  |  |  |  |  |  |  |  |
|  |  |  |  |  |  |  |  |  |  |
|  |  |  |  |  |  |  |  |  |  |
|  |  |  |  |  |  |  |  |  |  |
|  |  |  |  |  |  |  |  |  |  |
|  |  |  |  |  |  |  |  |  |  |
|  |  |  |  |  |  |  |  |  |  |
|  |  |  |  |  |  |  |  |  |  |
|  |  |  |  |  |  |  |  |  |  |
|  |  |  |  |  |  |  |  |  |  |
|  |  |  |  |  |  |  |  |  |  |
|  |  |  |  |  |  |  |  |  |  |
|  |  |  |  |  |  |  |  |  |  |
|  |  |  |  |  |  |  |  |  |  |
|  |  |  |  |  |  |  |  |  |  |
|  |  |  |  |  |  |  |  |  |  |
|  |  |  |  |  |  |  |  |  |  |
|  |  |  |  |  |  |  |  |  |  |

|  |  |  | | | | | | | |
| --- | --- | --- | --- | --- | --- | --- | --- | --- | --- |
|  |  |  | | | |  | | | |
|  |  |  |  |  |  |  |  |  |  |
|  |  |  |  |  |  |  |  |  |  |
|  |  |  |  |  |  |  |  |  |  |
|  |  |  |  |  |  |  |  |  |  |
|  |  |  |  |  |  |  |  |  |  |
|  |  |  |  |  |  |  |  |  |  |
|  |  |  |  |  |  |  |  |  |  |
|  |  |  |  |  |  |  |  |  |  |
|  |  |  |  |  |  |  |  |  |  |
|  |  |  |  |  |  |  |  |  |  |
|  |  |  |  |  |  |  |  |  |  |
|  |  |  |  |  |  |  |  |  |  |
|  |  |  |  |  |  |  |  |  |  |
|  |  |  |  |  |  |  |  |  |  |
|  |  |  |  |  |  |  |  |  |  |
|  |  |  |  |  |  |  |  |  |  |
|  |  |  |  |  |  |  |  |  |  |
|  |  |  |  |  |  |  |  |  |  |
|  |  |  |  |  |  |  |  |  |  |
|  |  |  |  |  |  |  |  |  |  |
|  |  |  |  |  |  |  |  |  |  |
|  |  |  |  |  |  |  |  |  |  |
|  |  |  |  |  |  |  |  |  |  |
|  |  |  |  |  |  |  |  |  |  |
|  |  |  |  |  |  |  |  |  |  |
|  |  |  |  |  |  |  |  |  |  |
|  |  |  |  |  |  |  |  |  |  |
|  |  |  |  |  |  |  |  |  |  |
|  |  |  |  |  |  |  |  |  |  |

|  |  |  | | | |  | | | |
| --- | --- | --- | --- | --- | --- | --- | --- | --- | --- |
|  |  |  |  |  |  |  |  |  |  |
|  |  |  |  |  |  |  |  |  |  |
|  |  |  |  |  |  |  |  |  |  |
|  |  |  |  |  |  |  |  |  |  |
|  |  |  |  |  |  |  |  |  |  |
|  |  |  |  |  |  |  |  |  |  |
|  |  |  |  |  |  |  |  |  |  |
|  |  |  |  |  |  |  |  |  |  |
|  |  |  |  |  |  |  |  |  |  |
|  |  |  |  |  |  |  |  |  |  |
|  |  |  |  |  |  |  |  |  |  |
|  |  |  |  |  |  |  |  |  |  |
|  |  |  |  |  |  |  |  |  |  |
|  |  |  |  |  |  |  |  |  |  |
|  |  |  |  |  |  |  |  |  |  |
|  |  |  |  |  |  |  |  |  |  |
|  |  |  |  |  |  |  |  |  |  |
|  |  |  |  |  |  |  |  |  |  |
|  |  |  |  |  |  |  |  |  |  |
|  |  |  |  |  |  |  |  |  |  |
|  |  |  |  |  |  |  |  |  |  |
|  |  |  |  |  |  |  |  |  |  |
|  |  |  |  |  |  |  |  |  |  |
|  |  |  |  |  |  |  |  |  |  |
|  |  |  |  |  |  |  |  |  |  |
|  |  |  |  |  |  |  |  |  |  |
|  |  |  |  |  |  |  |  |  |  |
|  |  |  |  |  |  |  |  |  |  |
|  |  |  |  |  |  |  |  |  |  |
|  |  |  |  |  |  |  |  |  |  |

|  |  |  | | | |
| --- | --- | --- | --- | --- | --- |
|  |  |  |  |  |  |
|  |  |  |  |  |  |
|  |  |  |  |  |  |
|  |  |  |  |  |  |
|  |  |  |  |  |  |
|  |  |  |  |  |  |
|  |  |  |  |  |  |
|  |  |  |  |  |  |
|  |  |  |  |  |  |
|  |  |  |  |  |  |
|  |  |  |  |  |  |
|  |  |  |  |  |  |
|  |  |  |  |  |  |
|  |  |  |  |  |  |
|  |  |  |  |  |  |
|  |  |  |  |  |  |
|  |  |  |  |  |  |
|  |  |  |  |  |  |
|  |  |  |  |  |  |
|  |  |  |  |  |  |
|  |  |  |  |  |  |
|  |  |  |  |  |  |
|  |  |  |  |  |  |
|  |  |  |  |  |  |
|  |  |  |  |  |  |
|  |  |  |  |  |  |
|  |  |  |  |  |  |
|  |  |  |  |  |  |
|  |  |  |  |  |  |
|  |  |  |  |  |  |

**Table S3.** Protein sequences deduced from the cds sequences belonging to different *gtf3a* ortholog genes, with length and deduced molecular weight. The sequences presented here were the ones used to produce the phylogenetic tree in Figure S1. Sequence underlined in yellow show the conserved initial sequence in all the oocyte specific Gtf3ab proteins of fish and the Xenopus oocytic protein. The sequence in blue shows the last of the C2H2 Zn finger domains of all the Gtf3as. In green the conserved transcription activation KRSLAS domain (KRSLAShLsGYPPK), necessary for transcriptional activation of 5S rRNA is shown. In teleostean proteins this is only found in Gtf3aa-s.

|  |  |  |  |
| --- | --- | --- | --- |
|  |  |  |  |
|  |  |  |  |
|  |  |  |  |
|  |  |  |  |
|  |  |  |  |
|  |  |  |  |
|  |  |  |  |
|  |  |  |  |
|  |  |  |  |
|  |  |  |  |
|  |  |  |  |
|  |  |  |  |
|  |  |  |  |
|  |  |  |  |
|  |  |  |  |
|  |  |  |  |
|  |  |  |  |
|  |  |  |  |
|  |  |  |  |
|  |  |  |  |
|  |  |  |  |
|  |  |  |  |
|  |  |  |  |
|  |  |  |  |
|  |  |  |  |
|  |  |  |  |
|  |  |  |  |
|  |  |  |  |
|  |  |  |  |
|  |  |  |  |
|  |  |  |  |
|  |  |  |  |
|  |  |  |  |
|  |  |  |  |
|  |  |  |  |
|  |  |  |  |
|  |  |  |  |
|  |  |  |  |
|  |  |  |  |
|  |  |  |  |
|  |  |  |  |
|  |  |  |  |
|  |  |  |  |
|  |  |  |  |
|  |  |  |  |
|  |  |  |  |
|  |  |  |  |
|  |  |  |  |

# NON TELEOSTEAN Gtf3a

**> Coelacanth (*Latimeria chalumnae*) Gtf3a ENSLACT00000016675, 298 aa, 34.87 kDa**

PKNFICSFEGCDASFNKAWKLDAHLCKHTGEKPFVCDYKGCGKGFTRNYHLTRHQLIHGGEKPFQCPNDGCNAAFSTKSNLKRHTENKHGNQDAPYVCDFEACGKAFKKHQQLKIHQCEHTNLLPFECDYEGCNKRFPVPSKLRRHKKIHKGYACEKEDCSFVGKTWTEYQKHLKDRHTEKFICELCNKTFKRKDFLKQHQKTHDQHREVFRCPHEGCGRTYTTTFNLQSHILSFHEERREYVCRQPGCGKAFAMKWTLNTHSISQDAFCLMYHLPKKRPPRPKRSLASRLSGYVPPK

# > Spotted gar (*Lepisosteus oculatus*) Gtf3a ENSLOCT00000005013, 373 aa, 43.07 kDa

MVRSHPCRGFRVVCSARIYGEHVCGKMEGDSVQFSSSSVTSSVIIPLSVFAMGETMGDPVKRFICSFPDCSASFNKAWKLEVHQYKHTGERPFVCDYEGCGKTFTRSFHLTRHQITHSGEKPFRCPVEGCDEVFPINCSLKRHVARIHEHQGKPYICKYEGCGKSFKKNNQLKSHEYEHTNILPFKCSFEGCDKRFLIPSKLKRHEKVHRGYPCKEDDCSFVGKNWTEYLKHKNALHQELLQCDQCSRTFKRNRLLQEHQRIHQEGRPILHCPREGCQRTYTTPFNLQSHILSFHEEQRPFACPHPGCGKAFAMRQSLQRHGVVHDPEKKKLKIPRPKRSMASRLSGFQPCKDEKKLAQLLQATSLESEEQST

**> Chicken (*Gallus gallus*) Gtf3a ENSGALT00000027621, 396 aa, 43.47 kDa**

MRAAAASAIAAPGGWDRHVTSLMAVEGAAGSESAPGGGSSSVGGAAVAGGSSDGSAPAPAGSGVGSAPAARSFICSFPGCSATFNKGWRLDAHLCSHTGARPYVCQYEGCGKSFTRDFHRTRHFLTHSGERPFECTAEGCNQKFGTKSNLKKHVQRKHENQQKLYSCNFEGCGKSFKKHQQLKVHLCQHTNEPPFKCNQEGCGKNFSTPNSLKRHKKTHEGYACKKENCSYIGKTWTELLKHNKESHTEPIVCTECSKTFKRKDYLKQHKKTHAAEREVCRCPREGCDRTYTTLFNLQSHILSFHEELKPFSCDHPGCGKVFAMKQSLARHAVHHDPEKKKLKAKRSRPKRSLASRLSGYIPPKTQPGKDVVVTECKTTDQPTENGIPTVEILTLQ

**> Human (*Homo sapiens*) Gtf3a ENST00000381140, 365 aa, 41.50 kDa**

LDPPAVVAESVSSLTIADAFIAAGESSAPTPPRPALPRRFICSFPDCSANYSKAWKLDAHLCKHTGERPFVCDYEGCGKAFIRDYHLSRHILTHTGEKPFVCAANGCDQKFNTKSNLKKHFERKHENQQKQYICSFEDCKKTFKKHQQLKIHQCQHTNEPLFKCTQEGCGKHFASPSKLKRHAKAHEGYVCQKGCSFVAKTWTELLKHVRETHKEEILCEVCRKTFKRKDYLKQHMKTHAPERDVCRCPREGCGRTYTTVFNLQSHILSFHEESRPFVCEHAGCGKTFAMKQSLTRHAVVHDPDKKKMKLKVKKSREKRSLASHLSGYIPPKRKQGQGLSLCQNGESPNCVEDKMLSTVAVLTLG

***> Xenopus laevis* Somatic Gtf3a P03001, 366 aa, 40 kDa**

MAAKVASTSSEEAEGSLVTEGEMGEKALPVVYKRYICSFADCGAAYNKNWKLQAHLCKHTGEKPFPCKEEGCEKGFTSLHHLTRHSLTHTGEKNFTCDSDGCDLRFTTKANMKKHFNRFHNIKICVYVCHFENCGKAFKKHNQLKVHQFSHTQQLPYECPHEGCDKRFSLPSRLKRHEKVHAGYPCKKDDSCSFVGKTWTLYLKHVAECHQDLAVCDVCNRKFRHKDYLRDHQKTHEKERTVYLCPRDGCDRSYTTAFNLRSHIQSFHEEQRPFVCEHAGCGKCFAMKKSLERHSVVHDPEKRKLKEKCPRPKRSLASRLTGYIPPKSKEKNASVSGTEKTDSLVKNKPSGTETNGSLVLDKLTIQ

> ***Xenopus laevis* Ovarian Gtf3a CAB51745, 344 aa, 38 kDa**

MGEKALPVVYKRYICSFADCGAAYNKNWKLQAHLCKHTGEKPFPCKEEGCEKGFTSLHHLTRHSLTHTGEKNFTCDSDGCDLRFTTKANMKKHFNRFHNIKICVYVCHFENCGKAFKKHNQLKVHQFSHTQQLPYECPHEGCDKRFSLPSRLKRHEKVHAGYPCKKDDSCSFVGKTWTLYLKHVAECHQDLAVCDVCNRKFRHKDYLRDHQKTHEKERTVYLCPRDGCDRSYTTAFNLRSHIQSFHEEQRPFVCEHAGCGKCFAMKKSLERHSVVHDPEKRKLKEKCPRPKRSLASCLTGYIPPKSKEKNASISGTEKTDSLVKNKPSGTETNDSLVLDKLTLQ

**TELEOSTEAN Gtf3a**

**> Asian_bony_tongue_Gtf3a_KPP62810, 365aa 41.53 kDa**

MAAAAAVQAPAPPLAAIARGSQSPSVRPLPVLVMGERLQDSHRLFICSFPDCGAAYNKSWKLDAHMCKHTGLRPFGCASKGCDKRFCTKYHLNRHALSHSGERPYRCTADGCSEAFTTSSNMKKHVARRHHNKEKLYVCDYEGCGREFKKHNQLKSHEFEHTNVLPYECNFEGCGRRFPVPSKLKRHEKVHKGYSCTEDGCSFQGKTWTEYQKHRKDQHQVQLQCPTCTKLFWNVWNFQQHQYVHQKERPVFCCPREGCQRTYTTAFNLQSHILSFHEELRPFSCSHPGCDKTFAMKQSLQRHSVVHDPEKKQQKKSRPKRSLASRLSGYQPKRSIPSEQSKLAVLLQDATLREANGAMALGD

>**Piraucu (*Arapaima gigas*) Gtf3a 348aa, 44.72 kDa (Vialle et al., 2018)**

MYSRKLAERSLYPGQRWYLPRDIQEFRRVNMEAAVAGGSCSTFVLPLSALVMGERLRDPSKLYICSFSDCGAVYNKSWKLDAHMCKHTGLKPFACESDGCDKRFCTKYHLSRHALSHSGQRSYRCTVEGCSEAFTTNTNMKRHVARRHQNREKLYVCEYEGCGREFKKHNHLKSHTFEHTNVLPYPCTFQGCEKRFLLPSKLKRHEKVHKGYFCTEDACNFHGKTWTEYQKHRREQHRVQLQCPSCERVFWDTWRLKQHQHTHEAKRTVFCCPREGCQRSYTTAFNLQSHILSFHEEQRPFSCPHPSCDKTFTLKQSLQRHSVVHDPEKKKQKKLRPKRSLASRLSGYQPKKSGLSEQGKLAVLLQDATLCDAGTGKTVGCNTR

**TELEOSTEAN Gtf3aa**

**> Cave fish (*Astyanax mexicanus*) Gtf3a XP_007235992, 450 aa 50.96 kDa**

FRLVVLQCTFEGCGKTFKKNNRLKIHECTHTQLLPYQCSHEGCERRFACPSKQKRHEKVHKGYPCSEEDCSFVGKTWTELLQHRKSHIAKVACDQCNRKFTDEWVLKQHQRVHGKERVVFRCPREGCQRSYTTAFNLQNHILSFHQEERAFTCPQPGCGKSFCMRQSLQRHSVVHDPERKKQKKPRPKRSLASRLSGYKPSKTRPAENGKPHKSTSSATSKSARSKSGRGQATSQSEPPEDIYDTRTSPELTKIRTNASHELNPTNADITPSSPPRMEGNETGQSEVTDSDSMIVLILEPLMLNSPAMSQSQPTEMYNTDISQSEPFRSETAMMSQSGPTQLESTMISQVEPLVTSQFESTISQLEPTKCKNVVISQLEPSKLENTVNSQLESSKFENTVNSQLESSEFENTVNSHLEPSKFENTVNSQLESSKFENIVNSQCDVIIRDG

> **Channel_catfish_(*Ictalurus punctatus*) Gtf3aa_XP017315713, 358 aa**  **41.45 kDa**

MFIMREFNKSFPRMFICSFPDCTAAYDKEWKLEAHLCKHTGVRPFGCEYAGCGKSFCSKSHLARHELTHTGEKPFSCGEDGCTQSFTTNFNLKKHISRKHKQEAKMYTCVFEGCGKSFKKNNLLKIHESTHTLQLPYECTYEGCDRRFANPSKRKRHEKVHKGYPCPAEDCSFVAKTWTELTKHRKEHKVRVQCEECKKTFRDQWFLKQHQHVHAEVRLVFLCPRDGCKRSYTTAFNLQSHILSFHEQQRAFSCPEPGCTKAFSMKLSLQRHSVVHDPERKKQKKPRPTRSLASHLSGYKTSKKRRKTPDLDDSPDSVTSQSETSKSDSNRAVISSEPVKDDVTWPCPMVLESSDQSQ

**> Cod (*Gadus morhua*) Gtf3aa ENSGMOT00000011273, 336 aa, 38.72 kDa**

MDTKRISERRYICSYPDCDARYNKQWKLDAHLCKHTGIKPFACEQSGCGKAFPSPYHLTRHQLTHSGMKPFPCTAAGCTDTFTTNTNMLRHFQRQHATDQKKYGCEVAGCGLVFKKNKQLNLHMCEQHTLLPPYQCSFEGCQMRFPCPSKQRRHEKVHNGYPCREEACVFTGKTWTELLKHRREAHQPVYPCDQCDKVFRKSWMLHQHQAVHADMRVVLKCPRAGCQRSFTTEFNLMSHIKSFHDELRPFACTHEGCGKTFAMKGSLTRHSVAHDPERRKIPKIRKPRPSRSLASRLSGVNPFKSARKTKELKDNTASGPHSPIKLFPLLQDTTLL

**> Fugu (*Takifugu rubripes*) Gtf3aa ENSTRUT00000017432, 339 aa, 39.19 kDa**

MEAGSEIQKRYICSFVGCQAAYNKQWKLDAHLCKHTGIKPFTCDRDGCGKSFCSQYHLARHDLSHSGVKPFRCSVDGCEDAFTTNANRDRHVSRVHSSDRKKYACRWDGCGLEFKKNKQLKAHMCEQHTQLPPYRCTHDGCEMRFAFPSKLKRHEKVHRGYPCSEEGCGFTGKTWTDYLNHRKEQHRRLLKCDQCSKEFRDSWFLQQHQRVHADTRVVLLCPHQGCGRSFTTVFNLESHIGSFHEELRPWVCTQVGCGKKFTMKQSLHRHSIVHDPQRKKLKKPRASQSLASKLSGYKETKTVLVKKKKEPESVRRSLQAAETHSSVELLSLLQDTSLQ

**> Medaka (*Oryzias latipes*) Gtf3aa XP_004084267, 358 aa 41.13 kDa**

METKTDVHKRYICSFPECSAAYNKQWKLDAHLCKHTGVKPFPCEQSGCGKSFCDRYHLARHELTHTGEKHFVCTIEGCEEAFSTRSNLNRHVSRKHSQERKTYVCTFDGCGLGFRKNNQLKLHLCEKHTQLPLYACTHEGCEMRFAVPSKLKRHEKVHKGYPCTEEDCTFTGKTWTELLRHKKESHQHVVKCEHCSKVFRDSWFLQKHLHVHDETRIVFKCPRDGCDRSYTTTFNLQSHIRSFHEGLRPFACSHSGCGKTFAMRQSLLRHRVVHDPEKKKQRKPRPKRSLASRLSGYSEAKGTIRKKPKQPKSQADSPKSNQMGSVELVSLLQDAALMCSSAVDAQELANPLTAPLTV

> **Northerns pike (*Esox lucius*) Gtf3aa ENSELUP00000040878 , 353 aa 40.82 kDa**

MELCTHVPRKTFICSFPNCHASYNKAWKLDAHICKHTGLKPFVCGQGGCDKSFCTKYHLARHQLSHSGERPYRCTVDGCTDAFTTNYNLKKHVNRKHSHEDRPLEYVCSFEGCGKAFKKNNQLKSHECQQHTNLPPFQCTFEGCGKRFTFPNKLRLHEKVHRGYPCEEDGCSFIGKTWTEYTKHRRDVHIVLLQCNECSRVFRDSWFLKQHQRIHAEERQVFVCPRERCQRSFTTPFNLQSHISSFHEELRPYICPHEGCGKTFAMKQSLQRHSVAHDPERKKQKKSRPKRSLASRLSGYKPKSCGPNPHTSSESVVSGQSEPANDVGSEPIELVALLQDSHLPHWVRNQITV

**> Platyfish (*Xiphophorus maculates*) Gtf3aa XP_005800526, 358 aa, 40.82 kDa**

METKAEPHRRFFCSFPDCSAAYNRQWKLDAHVCKHTGLRPHSCQHEGCRKSFCTPYHLARHELVHSGERPFRCSVEGCAEAFTTSTNRSRHVSRAHAREQKKYACAFQGCGQEFKKNKQLRAHMCEQHTQLPPYQCAHDGCQMRFSTPSKLKRHEKVHRGYPCAEEGCPFTGKTWTDLLKHRKEQHRVVLTCEHCSKVFRDSWFLQQHQRVHADTRVVYKCPREGCDRSYTTMFNLQSHVGSFHENLRPFVCTHDGCGRAFTMKRSLQRHSVVHDPEGRKPKKSRPKRSIASRLSGYREAKRVVCEKLPDPKRGLCHDKTEQPGSVVLVSLLQDTSLLCEPTVDTHGLADVMNPPLST

> **Red_bellied_piranha_Gtf3aa_XP017558412 , 511 aa** **58.77 Kda**

MVPSWWRQRQNEHGAVVSTWSGSPGLFWSLIFFFIMRQKSFICSFPDCQAAYDKQWKLEAHLCKHTGVRPFECQYEGCSKSFCTKSHLARHELTHSGQRPFKCTEEGCSQAFTTNSNLKKHISRKHRLQVKQYICSFEGCGKSFKKNNRLKTHEYTHSNLLPYECSYEGCERRFSIPSKRKQHEKVHKGYPCEAEGCSFIGKNWTEFTKHRRHHLVQCDQCKKMFNAQWKLKEHMRVHSAERVVFRCPKDSCQRSYTTAFNLQSHILSFHQEERAFTCTQPGCGKTFCMKQSLQRHSVVHDPERKKQFKKKPRPKRSLASRLSGYNPSRNRPVEASKPAKSTCVATNQSEQFKENNDQVVGLLQPVNEDSHVDSSEYTVTSTNPLTSASAEITQSWTHKTEEAEISQSESLNSDSVITLILEPLVLDSPAHSQMEPSEIENTVTIQSEPIDIEKTETIQSDSTEFANTVISQSEPIIFENTIVSQSELSNFENTVISQSEPTEFEKTVTIQ

> **Salmon (*Salmo salar*) Gtf3aa XP_013984796.1 355aa,**  **41.45 kDa**

MMELSQQVPRKSFICSFSDCHASYNKAWKLDAHICKHTGLKPFVCEHDGCDKSFCDKYHLARHQLSHSGERPFRCTVDSCTEAFTTNYNLKKHVNRKHNHDEEKLYEYVCSFEGCGKVFRKNNQLKSHECQQHTKLPPFQLYSCTFEGCEKRFTFPNKLRLHEKVHRGYPCEKDGCSFIGKTWTEYTNHRRDVHRALFQCNECNKVFRDTWFLKQHQRVHAEERQVFICPREGCQRSFTTPFNLQSHIGSFHEELRPYTCPHEGCGKTFAMKQSLQRHGVVHDPERKKQKKPRPKRSLVSRLSGYKPKNNSHLKPHAALQSESIVRGQSEQTNTVGSEPITLVSLLQDTVFCNSG

**> Stickleback (*Gasterosteus acuelatus*) Gtf3aa ENSGACT00000026241, 334 aa, 37.52 kDa**

MESKREPLRRYICSFAGCPAAYNRQWKLDAHLCKHTGVKPHACARCAKSFCTSYHLARHALSHSGEKPFRCPEDGCGEAFTTAANRARHVGRVHAPGRKRTYACRFEGCALEFRKNKQLKAHVCERHGPPAGHPCTHEGCAMRFAVPSKLRRHEKVHRGYPCADEGCGFTGKTWTEYLRHRKERHRPVLRCDQCDKSFRDSWFLQQHRRVHSETRVVLRCPRAGCERSFTTAFNLQSHVGSFHEERRPFACAHAGCGKTFAMKQSLRRHSVAHDPDKKKLAKARPKRSLASRLSGYGGTAAGKPAEESGPPGPVELVSLLQDTSLLCGPAVDTH

**> Tetraodon (*Tetarodon nigroviridis*) Gtf3aa ENSTNIT00000009239, 339 aa, 38.84 kDa**

MEANTEVSKRYICSFAGCTAAYNKQWKLDAHLCKHTGVKPFACDRDGCGKSFCSRYHLARHDLSHSGAKPFRCSADGCEEAFSTNANRARHVSRVHGSDRKTYACRFDGCGLQFRKNKQLRAHMCEQHTQLPPYLCPHEGCGMRFAFPSKLKRHEKVHRGYPCPEEGCGFTGKTWTDYLNHRKERHRRILKCDQCSKVFRDSWFLQQHQRIHADTRVVLLCPHVGCGRSFTKVFNLESHIGSFHEELRPYVCTHAGCGKKFTMKQSLHRHSIVHDPQRKKLKTPRASRSLASRLSGYKDTKAVLLKKKREPEPVRGAVQDAEPRTSVELVSLLQDTSLQ

**> Tilapia** (***Oreochromis niloticus***) **Gtf3aa ENSONIT00000026027, 340 aa, 39.78 kDa**

METKAESYKRYICSFSGCSAAYNKQWKLDAHLCKHTGVKPYSCERDGCSKAFCSKYHLARHELSHNGEKPFRCTVDGCAEAFTTNANRARHIGRIHCLEPRKYVCRFEGCGLEFKKNKQLKSHMCEQHTQLPPYQCIYEGCQMRFSFPSKLKRHEKVHRGYPCKEESCVFTGKTWTEYLKHRKEQHRVTLKCEQCSRVFRDTWFLQQHQRIHSEMRVVLKCPRDTCDRSFTTAFNLQSHISSFHEERRPFVCTHAGCGKTFAMNQSLQRHSVVHDPQRKKLKKPRAKRSLASRLSGYCETKRVVYKMQTEHAAHRQSSQEKADQPGPFELVSLLQDASLL

**>Turbot_(Scopthalmus maximus) Gtf3aa_AWP05158, 361 aa, 40.77kDa**

MPAGMEAKPQPHKRYICSFPGCAAAYNKQWKLDAHLCKHTGVRPHACPRDGCGKSFCSPYHLARHELSHSGERLFRCTADGCAEAFTTNSNRSRHVGRAHGREQKTYACAFDGCGLEFRKNKQLRAHVCERHAQLPPYPCAHEGCHMRFAFPSKLRRHEKVHRGYPCEEEGCDHTAKTWTEHLRHRKERHRPVLRCDQCSKVFGDSWFLQQHRRVHSEVRVVLKCPREGCSRSFTTTFNLQSHISSFHEELRPFACAHAGCGKTFAMKQSLRRHSVVHDPERRKLRKPKPGRSIASRLSGFREAKVCEKRRGPEPPGSRGSEPPGPVELVSLLQDASLLCSPAAAVDTHGLTSALATPLAV

**> Zebrafish (*Danio rerio*) Gtf3aa ENSDART00000105925, 367 aa, 42.62 kDa**

MTMDETNANVDQLGEIFICSYPECHAYYNREWKLQAHLCKHTGERPYKCKYKKCSKSFCTKHHLTRHVLTHTGEKPYRCVEDGCKEGFTTNSNLQKHISRIHRQETKQYICTFEGCGKAFKKNNQLKTHECTHTQLLPFLCTQEGCGRRFSQRGKLKRHEKVHKGYSCETEGCSFVAKNWAEMTNHKKVHIVRVQCDQCQKTFRDSWFLKQHQHVHSEERLVFHCPRDGCTRPYTTAFNLQSHILSFHEQQRSFICAHPGCGKAFSMKQSLQRHGVVHDPEKKQMKPRPKRSLASRLSGYKSKKTRQTKTETSAPQISQSENPTHPQSDNQLHCLSSETLPSSHLEPVKSICSPANPVMHLLEPFLV

**TELEOSTEAN Gtf3ab**

# > Cave fish (*Astyanax mexicanus*) Gtf3ab ENSAMXT00000014879, 329 aa, 37.76 kDa

MGERLKDPNKSFACLYSDCKASFSKSWKLEAHYCKHTGVRPFACDSCTKSFCTRYQLTRHQLSHRGETPHLCSVDGCAEAFSTIGRLKNHVSRAHEKEQKRYVCNYEGCGKEFCKKRQLKTHHCEHTNELPYECKFEGCGKKYAASKALKKHEKMHNGYPCAEEGCLFKGKTWTEYQAHRKAEHREILQCGDCKKVFYVAWFLLKHKQFVHSGERRVFKCTKEGCEKTYTTNFNLQNHILSFHEGKRPFICSHAGCGKAFAMEKSLKRHGVAHDPNKKKMQVNILEKQLTPQKTKRKNVPSKAKASDASALSARLKNVSLNKDASQNNP

> **Channel_catfish_(*Ictalurus punctatus*) Gtf3ab_NP00187205** 322aa, 37.33kDa

MGERFKDPAKNFVCSFLNCKASFSKAWKLEAHYCKHTGLRPFACDRCDKTFCTRCQLTRHNLSHSGKKPYQCLEDGCSESFISTAGLKNHVERVHQHKEKHYVCDYEGCAKEFRKKKQLRSHKCEHMNQLPFECQYEGCGKKYTTSKKLQKHEKVHDGYPCAEEGCDFQGRMWTEYQAHRKAAHREALQCDSCAKVFHKAWFLKKHKLFVHLGVRRVFKCTKEGCQKTYTTHFNLQNHILSFHEGIRSFICPHDGCGKAFAMEGSLKRHAVVHDPQKKKLQKKTKRGRKKKLEPKTNVSDDSELPAQLHGLSLNTSTSQNNP

# > Cod (*Gadus morhua*) Gtf3ab ENSGMOT00000016779, 299 aa, 34.16 kDa

MGERIHIKKSFICSFHDCSASFSKSWKLEAHNCKHTGLKPFSCDDCDKNFCTRYQLTRHQLNHSGDRPHKCQADGCGEAFVSQSSMKNHMDKSHHNEGKPFKCNHQGCGKDFSKRYQLKAHVYEHTKVLPFHCTVTGCTREFPSRGSLDHHKKVHQGYPCEEDGCPFQGKTWSAYQTHKKEHRVKLPCDKCKKQFNNGRFLLLHKRHVHLGVKKELACPHKCGKSFTRQFHLESHVLLEHEGVRAFGCAFPGCGKRFAMKESLWRHGVVHDPKRKAVKAKPTSAEGCMLAAKLAKIRGS

# > Fugu (*Takifugu rubripes*) Gtf3ab ENSTRUT00000012642, 327 aa, 38.11 kDa

MGERLQSQKSFVCTFFDCKAKFSKLWKLEAHLCKHTGLKPFSCESCDKSFCTRYQLTRHELNHSGERPHKCPAEGCPEAFVTHSSMKNHMGRVHHQRERPYQCDHLGCVKSFNKRNQLKAHQGEHQNVLPFHCSLKGCSREFPTHGKLKHHERVHAGYVCETNACPFEAKTWTEYLKHRKKHQAKVPCQRCQKLFNNAWFMHQHELRVHFGERRKLLCPKKGCNKEFTHRFNLDSHIQGDHEGKRSFGCAYAGCGKSFVMKESLWRHEVVHDPAKKKVKKLRPKRNQPWRLALRRKLAAAANQAETSKLAAKLRDTTLERQSSEGNV

> **Northern pike (*Esox lucius*) Gtf3ab: ENSELUP00000014039, 318aa 36.9 kDa**

MTVLKPTISPRKATFNKSWKLDAHLCKHTGLKPFSCKNCDKRFCTRYELTRHELSHSGERPYKCQAEGCAADFVTHASMKNHMIQVHQLQEKHYKCDHEVCGKEFRKKSKLKTHKMEHKQLLPFQCDIEDCKKQFAAPGQLKRHGKVHQGYPCAVEDCPFQGKTWSEYQKHRKAVHRVKLQCDSCSRVFLEAWFLKQHQLRIHSDVPKKVFQCSDAECRKTFTKRFNLENHQLSEHEGKKAFCCAHEGCGKSFAMQESLRRHQVVHDPLKKKLQKRHPKKGKSSQRKTKTESGTASTQAETSELAARLRNTSLDDSTP

# > Medaka (*Oryzias latipes*) Gtf3ab ENSORLT00000011034, 266aa, 31.31 kDa

MGEKLQSQKTYSCSFSDCKSTFGESWKLEAHMCKHTGLKPFSCGNCDERFCTQHQLTRHELSHSDEKPIMKNHVSNTQELQKKHFNCHMRVWKEFHKRNQRKLHVSINLCYPFRDCRFPGCAKEFLSNGKRRHHERVHQGYSCNNEVCPFQAKTWTELQKHKKEHEVKVQCGACQKLFSNKWFLHLHELRVHSGEKKLLSCPREGCDRKFMRRFNLESHVLGEHEGKKPFICAHAGCGKSFAMKESLRRHGAVHDPAKKKLKVKLR

# > Platyfish (*Xiphophorus maculates*) Gtf3ab ENSXMAT00000009903, 318 aa, 36.86 kDa

MGERLQSQNAYVCSFSDCNATFRKSWKLEAHLCKHTGLKPFSCESCEKSFCARYELTRHERVHSGEKPHTCPMDGCLEAFAKNATMKNHITRVHQHQEDRYKCDYEGCGKDFSKKKQLKAHKCEHGEPLAFHCTFNGCGKDFPSREKLKHHEKVHQGYPCSFDLCPTLSKTWTEYLKHRAQHREKLVCEKCNRLFNNSWFLRLHELRAHSGEKRYFLCPREGCNRKFTRRVKLESHVLGDHEGKKPFSCAYPGCGKSFALKESLWRHGVVHNPAKRELKKRKPKKDKPPQVAQEATRSAADDQETGKLAAKLHSTTLE

**> Red_bellied_piranha_Gtf3ab_XP017577611 , 316 aa, 36.22 kDa**

MGERLKDPNKSFACLFSDCKASFSKSWKLEAHYCKHTGLRPFACDGCTKSFCTRYQLTRHQLSHSGERPYLCSVDGCAEAFSTSASLKNHVGRVHQNKAAQYVCNYEGCGKEFYKNKQLRAHQSEHSNLLPFECHFEGCGKKYATSQTLKRHEKVHKGYPCAEEGCPFKGRTWTEYQAHRKVEHREILQCDGCKKVFYEAWFLQKHKQFVHSGERRMFKCTKEGCQKTYTTHFNLQNHILSYHEGKRSFICSREGCGKAFAMEQSLKRHAVVHDPQKKKKKTKLKNLPKTKVSDASELTSRLQNVSLNQSASQK

**> Salmon (Salmo salar) Gtf3ab XP_014036981.1 323 aa, 37.4 kDa**

MGERIQVHRSFICSFVNCNATFNKSWKLDAHLCKHTGLKPFSCENCDKSFCTRYQLTRHGLSHSGEKPYTCQAAGCSEAFVTYASMKNHMARVHQHQEKYYKCDHVHCGMEFNKKNQLKTHKIKHTQLLPFQCIFEGCKREFAAPGQLKRHEKVHQGYPCAVEDCPFQGKTWSEYQKHRKAVHRIELQCDSCSRMFLEAWFLKQHQLRVHSGAPKRVFQCSDAGCEKTFTTHFNLENHVVSDHEGKMAFSCTHEGCGKHFAMQESLRRHRVVHDPERKKLQKVHPKKRKPLLRKTELGTASTQVETSRLADQLHTASLGYSTS

# > Stickleback (*Gasterosteus aculeatus*) Gtf3ab ENSGACT00000004076, 324 aa, 37.09 kDa

MGERLQSPKMYICSFSDCKAAFSKSWKLEAHLCKHTGLKPFSCENCDQSFCTRYQLTKHELHHSGEKTHKCPADGCSRVFVTKGSMKNHVARAHQQQEKQYPCDHQGCGKGFNKRNQLKAHKCEHLQILPFPCTISGCTKALPTHGKLKRHEKVHRGYQCEQEACPFQGNTWTEYLQHRKEHKVKVQCGECKKLFNNAWFLHQHGLRVHAGEKPQQQLLCPKEGCGKRFTRPFNLESHVLGDHEGKKPFSCVYAGCGKSFAMKESLWRHGVVHDVTKKKLKKVPKKKTWVKAVQVRQANQAEANKLAEKLHNATLKDDRGSRSP

# > Tetraodon (*Tetraodon nigroviridis*) Gtf3ab ENSTNIT00000015893, 318 aa, 37.03 kDa

MGERLQSQKNYVCTFLDCGAKFSKSWKLEAHLCKHTGLKPFSCESCSRSFCTRYQLTRHQLNHSGERPHKCPVEGCPEAFVTHASMKNHMARIHRPQEKAYQCDHWGCTKSFNKRNQMKAHQGEHQNLLPFHCSFDGCVREFPTHGKLRHHKRVHAGYVCETDACPFEAQTWTEYLKHRKKHQDKVPCSRCQKLFSNAWFLHQHELRVHSGEKRKLRCPRKGCNKEFTRHFNLESHLQGEHEGKRPFSCAHAGCGKSFAMKESLWRHGVVHDPAKRKVQKLRPKRNQPWRLALRRKLAAAANQAEASELAAKLRNTTL

**> Tilapia** (***Oreochromis niloticus***) **Gtf3ab ENSONIT00000017785, 326 aa, 37.97 kDa**

MGERLQKTYVCSFSDCKATFSKPWKLDAHLCKHTGLKPFSCESCDKSFCTRYQLTRHELSHSGEKPHKCPADGCSEAFVRNASLKNHIARVHQQQEKRFQCDHQGCEKDFSKRNQLKAHQCEHQESLPFHCSLTGCTREFLTLKKLKHHEKMHEGYPCETDGCPFQGKTWSDYLKHRKEHKDKVLCGHCNKLFSNFWFFRLHELRVHSGEKRTFPCPKEGCEKKFTRRFNLESHVLGDHEGKKPFSCAVPGCNKSFAMKESLWRHGVVHDPAKKKLKKLHPKKNRPMAQRDKQHTFKHMYKTPAAIAGLRLIQLDTALVLQNISIK

> **Turbot_(Scopthalmus maximus) Gtf3ab_AWP20656, 323 aa,** **37.32 kDa**

MGERLQSPKSYVCSFFDCDATFSKSWKLEAHLCKHTGLKPFSCENCDKSFCTRYQLARHELNHSGEKPHKCLADGCSEAFVTNASMKNHMARVHQQQEKRYKCDYQGCAKDFNKRNQLKSHKGEHEQLLPFHCTFSGCTKEFPSHGKLQHHKKVHEGYPCEAEACPFQGKTWTEYLKHRKEHKVKVLCGECKKRFNNTWYLHQHELRVHSGERRMLSCPREGCDKKFTGRFSFENHVLGDHDGKRPFSCAYAGCGKSFAMKENLWRHGVVHDPAKKKLKKLHPKKNQPWRKALQVKLAAAANQADANKLAAKLRNTALEDKKS

# > Zebrafish (*Danio rerio*) Gtf3ab ENSDART00000105925, 318 aa, 37.15 kDa

MGERIQDPNKHFTCTFADCKATFSKLWKLEVHYCRHTGLKPFACGDCEKTFCTRYQLTRHQLSHSGEKPYLCSVSGCSAAFSTPGSLRNHIAQVHDNKVRHYVCNYQGCAKEFHKKKQLKTHLCEHTNELSFKCDHEKCNNKFASPKALKRHRKLHEGYPCGEENCNFKGNTWSEYLKHRRTAHRVNLPCNQCKKVFHKVCFLQMHKKFVHSGERRMFKCTREGCQKSYTRRFNLENHVLDFHEGKRDFTCHFTGCDKAFAMEESLKRHFVVHMPQKTKPQKPKVKPKRKKTSKAKTSDAAKLSEHLQKVSLTKTPLP
